# Contrast independent biologically inspired translational optic flow estimation

**DOI:** 10.1007/s00422-022-00948-3

**Published:** 2022-10-27

**Authors:** Phillip S. M. Skelton, Anthony Finn, Russell S. A. Brinkworth

**Affiliations:** 1 Centre for Defence Engineering Research and Training, College of Science and Engineering, Flinders University, 1284 South Road, Tonsley, South Australia 5042 Australia; 2 Science, Technology, Engineering, and Mathematics, University of South Australia, 1 Mawson Lakes Boulevard, Mawson Lakes, South Australia 5095 Australia

**Keywords:** Bioinspired, Optical flow, Robotics, Computer vision, Time to impact, Contrast dependence

## Abstract

The visual systems of insects are relatively simple compared to humans. However, they enable navigation through complex environments where insects perform exceptional levels of obstacle avoidance. Biology uses two separable modes of optic flow to achieve this: rapid gaze fixation (rotational motion known as saccades); and the inter-saccadic translational motion. While the fundamental process of insect optic flow has been known since the 1950’s, so too has its dependence on contrast. The surrounding visual pathways used to overcome environmental dependencies are less well known. Previous work has shown promise for low-speed rotational motion estimation, but a gap remained in the estimation of translational motion, in particular the estimation of the time to impact. To consistently estimate the time to impact during inter-saccadic translatory motion, the fundamental limitation of contrast dependence must be overcome. By adapting an elaborated rotational velocity estimator from literature to work for translational motion, this paper proposes a novel algorithm for overcoming the contrast dependence of time to impact estimation using nonlinear spatio-temporal feedforward filtering. By applying bioinspired processes, approximately 15 points per decade of statistical discrimination were achieved when estimating the time to impact to a target across 360 background, distance, and velocity combinations: a 17-fold increase over the fundamental process. These results show the contrast dependence of time to impact estimation can be overcome in a biologically plausible manner. This, combined with previous results for low-speed rotational motion estimation, allows for contrast invariant computational models designed on the principles found in the biological visual system, paving the way for future visually guided systems.

## Introduction

Researchers have long been interested in how insects move so freely through complex environments. Of particular interest are those that use their visual perception systems as the primary source of sensory input during locomotion (Collett and Land 1975; Srinivasan et al. 1996; Borst and Haag 2002; Ruffier et al. 2003; Serres et al. 2008; Mauss and Borst 2020).

While there is growing neurophysiological support for the biological processes used by insects to tackle various aspects of this complex problem, there still exists a disconnect between the biological knowledge obtained by neurophysiologists and the technological capabilities of systems implemented by roboticists and engineers (Franceschini et al. 1992; Srinivasan et al. 1999a, b; Cuntz et al. 2007; Schwegmann et al. 2014; Bertrand et al. 2015; Li et al. 2016; Medathati et al. 2016; Meyer et al. 2016; Lecoeur et al. 2018).

This paper investigates one possible solution for overcoming the contrast dependence of a time to impact estimator that is inspired by insect biology. Using methods of adaptation that are biologically plausible, an existing algorithm for correcting for rotational errors during inter-saccadic motion (Skelton et al. 2019) has been adapted to consistently estimate the time to impact during the periods of inter-saccadic translation. Research into biological vision systems and their implementation as engineered solutions has shown that these models often contain a large number of parameters that can be tuned to produce different operating behaviours depending on the situation tested (Franceschini et al. 1992; Ruffier and Franceschini 2005; Serres et al. 2008; Brinkworth and O’Carroll 2009; Schwegmann et al. 2014). In the research reported here, this is the case: 42 parameters are used to specify the operating behaviour of the proposed algorithm. The complex task of tuning this parameter set followed a similar approach to that used to tune a rotational optic flow model (Skelton et al. 2020) and will be reported in-depth elsewhere.

## Related work

Humans perceive self-motion from visual inputs, or ‘egomotion’ as it is known (Dodge 1923; Warren 1976). It was found that humans are capable of detecting and relatively quantifying their motion vector (rotational and translational), from very sparse amounts of data. However, it was a requirement that there be contrast changes in the visual inputs for egomotion detection to occur (Lappe and Rauschecker 1993).

This interest in how the world is perceived also extended to other aspects of biology, where flying insects in particular have been a large source of research over the years, with the visual pathways of species such as the fruit fly *Drosophila melanogaster* (Fry et al. 2003; Maimon et al. 2008), the hoverflies *Syritta pipiens* (Collett and Land 1975) and *Eristalis tenax* (Straw et al 2006; Nordström et al. 2008), the blowfly *Calliphora vicina* (Hateren and Schilstra 1999; Schilstra and Hateren 1999; Borst and Haag 2002; Cuntz et al. 2007; Borst et al. 2010; Kern et al. 2012; Ullrich et al. 2015), the honeybee *Apis mellifera* (Srinivasan et al. 2000; Barron and Srinivasan 2006; Boeddeker et al. 2010), the tropical sweat bee *Megalopta genalis* (Warrant et al. 2004), the dragonfly *Hemianax papuensis* (Stange et al. 2006; Shabayek et al. 2018), and the hawkmoths *Manduca sexta* (Zhu et al. 2020) and *Macroglossum stellatarum* (Stöckl et al. 2019) all being studied. From these widespread neurophysiological investigations into biological vision systems, there are some key findings that are of interest to the field of robotics.

Firstly, it has been shown that both bees (Srinivasan et al. 1996; Barron and Srinivasan 2006) and flies (Collett and Land 1975; Borst and Haag 2002; Maimon et al. 2008) possess relatively simplistic visual systems compared to humans. While humans have both a small region of high acuity, known as the fovea, which is the point of fixation, and a large region of low acuity peripheral vision surrounding the fovea (Campbell and Green 1965; Williams and Coletta 1987; Intriligator and Cavanagh 2001), insects typically have a wide field of view with relatively low acuity, and no specialised fovea (Collett and Land 1975; Franceschini et al. 1992). However, it has also been shown that the males and females of certain species possess slightly different visual systems, known as sexual dimorphism (Nordström et al. 2008). For example, male flies have regions of higher visual acuity specifically developed for the detection and tracking of small objects, and this dimorphism is caused by different behavioural requirements between the sexes, such as males chasing females (Hardie 1986; Hornstein et al. 2000). Additionally, it has been shown that optic flow is used to perform obstacle avoidance or landing manoeuvres, and this can be thought of as the perceived time to impact of the insect to the environment (Krapp et al. 2001; Tammero and Dickinson 2002). Without precisely knowing ones’ own absolute velocity (egomotion), nor the absolute velocity of obstacles within a dynamic environment (e.g. predator or prey), or even the distance to objects, it is possible to calculate the time to impact that object represents by gauging the perceived relative optical flow. By exploiting motion parallax, objects at a closer distance will have a shorter time to impact compared to objects further away—assuming constant velocity—and will be associated with higher levels of optic flow.

The second finding is the visual behaviour known as saccadic motions, or saccades. Saccades are extremely rapid gaze fixations that typically last for approximately 40 milliseconds in humans (Castet 2009) and 40–50 milliseconds in flies (Schilstra and Hateren 1999; Fry et al. 2003). This behaviour has also been identified in non-flying insects, such as the ant *Formica rufa* (Lent et al. 2010) and the praying mantis *Sphodromantis viridis* (Rossel 1996), as well as several species of aquatic life (Land 1999). The purpose of saccades, in both humans (Robinson 1964; Becker and Fuchs 1969) and insects, is to separate the rapid high-speed rotational velocities from the low-speed rotational velocities and the translational velocities. This separation primarily occurs to allow for the accurate encoding of low-speed rotational velocity predominantly found during translational movements (Corthals et al. 2019), and the ability for motion sensitive neurons to interpret information about the spatial layout of the environment during the inter-saccadic translatory movements without significant contamination by rotational egomotion (Lindemann et al. 2008). Saccades are also used to overcome large optical displacements typically found during prey-tracking (Egelhaaf and Kern 2002). Saccades are not only both highly accurate and reproducible (Clark and Stark 1975; Lee et al. 1988), but are contextually dependent (Patla and Vickers 1997; Tomsic and Theobald 2017). This separation between rotational and translational optic flow calculation is reflected in the separation between research into the horizontally (yaw) selective cells, and the translational selective cells (Longden et al. 2017; Strother et al. 2017; Lecoeur et al. 2018).

Even with the comparatively less complex visual systems compared to humans (Dyer and Griffiths 2012), flies and bees are still capable of achieving linear velocities of 1.2 m/s and accelerations of 10 m/$\hbox {s}^2$ (Schilstra and Hateren 1999), as well as rotational velocities of 1000 $^\circ$/s and accelerations of 2000 $^\circ$/$\hbox {s}^2$ (Clark and Stark 1975). These manoeuvres are achieved during high-speed saccadic motions, while performing real-time complex tasks such as localisation, navigation, object detection, and object avoidance (Borst and Weber 2011).

Spatial pooling for optic flow estimation is a well-studied area, and it has been shown that several insects employ spatial pooling on their receptive fields for both noise suppression and object saliency. Both of these are thought to be important in the context of estimating time to impact as it is desirable for small targets (e.g. obstacles) to be more prominent features, and noise can be misconstrued as a small target (Egelhaaf and Warzecha 1999; Egelhaaf et al. 2002; Borst and Haag 2002).

Optic flow techniques have been used extensively in research for bridging the gap between biological capabilities and technological implementations (Horn and Schunck 1981; Franceschini et al. 1992; Barth et al. 2012; Kern et al. 2012). However, egomotion estimation techniques are largely dependent upon controlled environments, from alternating contrast bars in honeybee navigation studies (Srinivasan et al. 1996) and spatially perfect contrast images in ground robots (Mizutani et al. 2003) to well-lit daytime image sequences for computing the optic flow of autonomous landing of unmanned aerial vehicles (Thurrowgood et al. 2009; Shoemaker et al. 2011; Thurrowgood et al. 2014).

Although existing techniques that employ simple models of biological vision have largely ignored contrast dependency, research has shown that employing ‘elaborated’ models—that is models that more accurately represent the dynamic nonlinearities inherent within the biological system—can produce velocity estimates that are more environmentally invariant (O’Carroll et al. 2007; Brinkworth and O’Carroll 2009; Babies et al. 2011; Schwegmann et al. 2014; Ullrich et al. 2015) and resilient to noise (Brinkworth and O’Carroll 2010). These models, such as the fully elaborated, which rotational optic flow model proposed by Brinkworth and O’Carroll (2009)), which are more accurate in their representation of biological systems, have been shown to be synergistic in nature. That is, the performance of the system as a whole is greater than the sum of the individual components that make up that system (Brinkworth and O’Carroll 2009).

The foundational mathematical model for optic flow estimation by insects, the Hassenstein–Reichardt elementary motion detector, is a correlation-based technique. This results in high levels of contrast dependence as the larger the contrast, the larger the difference between samples. It is well known that the visual systems of a wide variety of animals adapt to the environment in which they are in, primarily by contrast, and has been shown to occur in flies and bees (Mileva et al. 2007; Babies et al. 2011; Bahl et al. 2015; Arenz et al. 2017; Wienecke and Clandinin 2020), as well as humans (Blackwell 1946; Gibson 1950). As the exact biological process for contrast adaptation is unknown, several potential applications of feedback and feedforward adaptation have been proposed with some neurophysiological support when dealing with rotational motion (Carandini and Heeger 2012, 2013; Drews et al. 2020).

While there have been attempts at overcoming the contrast dependence of an algorithm based upon Hassenstein–Reichardt detectors (Hassenstein and Reichardt 1956) in a translational setting, these have largely been applied locally to a specific scene, or have failed to incorporate the supporting elaborations that have been neurophysiologically proven to exist within the visual pathways of many insects (Rajesh et al. 2002; Straw et al. 2008). This work aims to overcome both of these shortcomings by operating with a complex dataset, and including biologically inspired elaborations to overcome the contrast dependence of the algorithm.

There is growing neurophysiological support for contrast adaptation being handled by separable ON–OFF pathways in the neural systems of insects such as the *Drosophila melanogaster* (Joesch et al. 2010; Kohn et al. 2021). While this has been shown to be prevalent in motion estimation during both rotational (Borst et al. 2020) and translational (Fu and Yue 2020) motion, these experiments still tend to deal with idealised contrast grating patterns or simplified visual inputs.

## Methods

The research undertaken in this paper has two aspects: the experimental equipment used, and the new computer vision algorithm developed. This section describes both of these in-depth.

### Experimental set-up

The camera used in this research was an Imaging Development Systems (IDS) GmbH UI-3060CP-M-GL Rev.2. This sensor is capable of capturing 12-bit monochrome images, left padded in 16-bit containers, at a resolution of $1936 \times 1216$ at up to 166 frames per second. All camera interfacing was achieved using the IDS C++ SDK. Coupled to this camera was a Palnon PAL-25G3817-27C omnidirectional annular lens, providing a full 360$^\circ$ FOV around the azimuth, with an altitude FOV ranging from $-15^\circ$ to $+38^\circ$ relative to the horizon. This lens has a fixed focal length and a fixed iris.

**Fig. 1:**
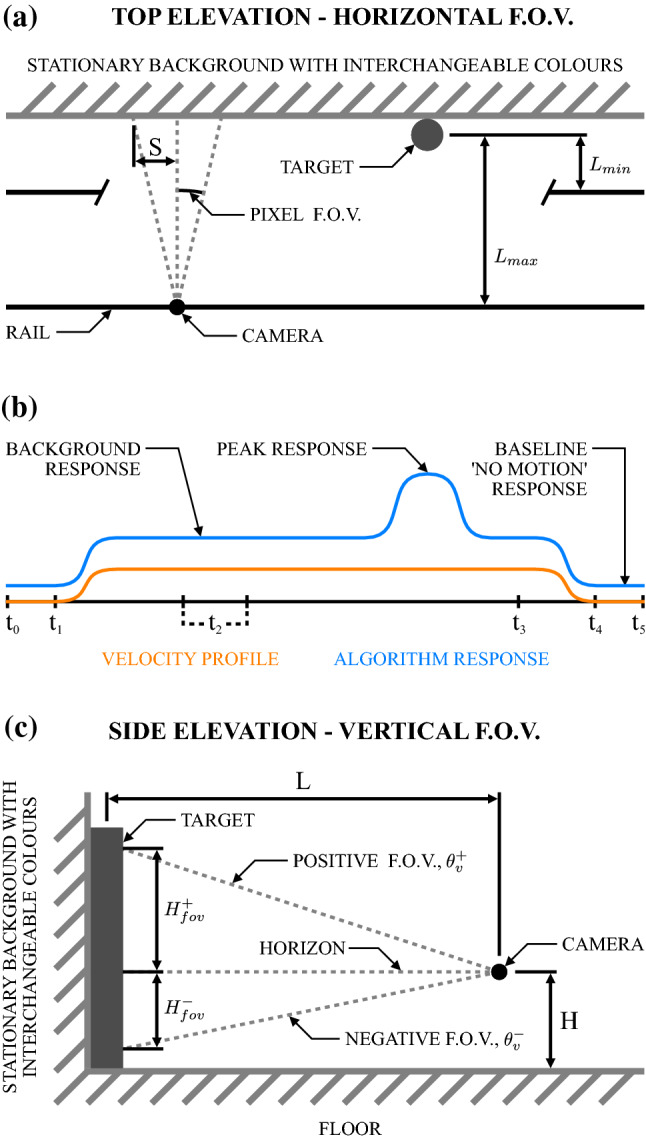
Diagrammatic representation of experimental set-up: **a** Top elevation. The camera rail was situated parallel to the wall, with the camera translating from left to right. The distance between the optical centre of the camera and the target was given as *L*. Exact target location along the x-axis of the rail was not critical as the scene was over-sampled, and was approximately 800 mm from the left extent in practice. The field of view (FOV) of each pixel is given (not to scale), with the resultant spatial sampling width given by *S*. **b** A characteristic velocity profile and algorithm response profile, with indications of where background, peak target, and baseline (no motion) responses were sampled. Full recordings occurred between times $t_0$ and $t_5$, where the end point $t_5$ was variable based on the velocity (spatial distance along the rail was equal for all recordings). The periods $t_0$ to $t_1$ and $t_4$ to $t_5$ were equal to 1 second of no motion. Recordings were clipped in post-processing to between $t_2$ and $t_3$, where $t_2$ was variable to provide equal adaptation time for the algorithm(s) before the target entered the receptive field. No motion and background responses were taken from the full recordings, with peak response taken from clipped recordings to reduce parameter tuning times. **c** Side elevation. The height of the optical centre of the camera relative to the floor was denoted by *H*. The positive and negative FOV’s of the lens used were unequal with +38$^\circ$ and -15$^\circ$ of FOV, respectively

For the purposes of this research, time to impact is defined as if the observer (e.g. insect, camera, etc.) was capable of instantaneous omnidirectional motion. While this is not biologically plausible, it simplifies the degrees of freedom of the problem and allows the focus to be on understanding how the motion energy is dependent upon the contrast, without the need to consider the angular content of the optic flow vector.

The experimental set-up can be seen in Fig. 1. Two factors were critical: The distance of the optical centre of the camera from the optical centre of the target, *L*; and the height of the optical horizon of the camera from the floor, *H*. To ensure that the target was always within the vertical FOV, the height of the optical centre of the camera needed to be greater than or equal to the spatial distance resolved by the negative vertical FOV at a given distance to target *L* (Fig. 1c).

Solving the system requirements parametrically, the maximum distance that the camera could be from the target, $L_\text {max}$, is:

1 $$\begin{aligned} L_{\max } = \frac{H_t}{\tan \theta _v^- + \tan \theta _v^+} \end{aligned}$$

where $H_t$ is the height of the target, and $\theta _v^+$ and $\theta _v^-$ are the positive and negative vertical fields FOV’s of the lens, respectively. Calculating the spatial distance resolved by the vertical FOV is given by:

2 $$\begin{aligned} H_\text {fov}^+= & {} L \tan \theta _v^+ \end{aligned}$$

3 $$\begin{aligned} H_\text {fov}^-= & {} L \tan \theta _v^- \end{aligned}$$

where $H_\text {fov}^+$ and $H_\text {fov}^-$ are the spatial distances resolved by the positive and negative FOV’s, respectively. The minimum and maximum heights of the optical centre of the camera, $H_\text {min}$ and $H_\text {max}$, respectively, are then:

4 $$\begin{aligned} H_\text {min}= & {} H_\text {fov}^- \end{aligned}$$

5 $$\begin{aligned} H_\text {max}= & {} H_t - H_\text {fov}^+ \end{aligned}$$

**Table 1:** Resultant methodological parameters

Parameter	Unit	**Ideal**	Practical	Equation
$\theta _v^+$	$^\circ$	–	$+38$	
$\theta _v^-$	$^\circ$	–	$-15$	
$L_\text {min}$	mm	–	200	
$L_\text {max}$	mm	$\sim$1144	1000	(1)
$H_{fov}^+$	mm	$\sim$894	$\sim$782	(2)
$H_{fov}^-$	mm	$\sim$306	$\sim$268	(3)
$H_\text {min}$	mm	$\sim$306	$\sim$268	(4)
*H*	mm	–	$\sim$350	
$H_\text {max}$	mm	$\sim$306	$\sim$418	(5)

The ideal values are the mathematical boundaries for the given parameter, whereas the practical values are those that could be, or were, used in real-world implementations while still satisfying the requirements of the system

The results of these equations can be seen in Table 1, where ideal and actual/practical values are shown. To account for any angular misalignments that were inadvertently introduced during the experimental set-up, and to provide equispaced data points, the maximum distance of the camera to the target, $L_\text {max}$, was 1000 mm, compared to the 1144 mm ideal value. While this provided a range of camera heights that would satisfy the requirement for the target to remain within the FOV of the camera, $H \in {\mathbb {N}} \ [H_\text {min}, H_\text {max}]$, a value of $H=350$ mm was used as this resulted in increased stability and repeatability on the supporting tripods of the rail. The minimum camera distance, $L_\text {min}$, was set to 200 mm due to the physical limitations of camera target geometry and experimental platform design.

**Fig. 2:**
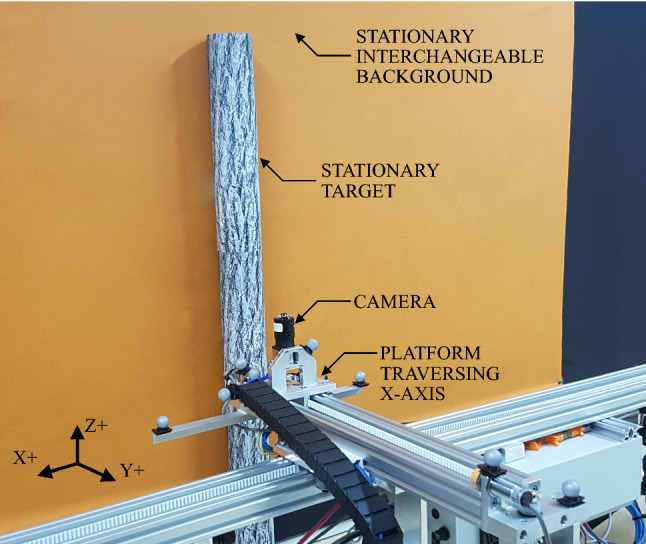
Experimental set-up constructed for this research with interchangeable backgrounds. The primary longitudinal axis, *x*, had 2600 mm of encoded linear travel (not all shown). The shorter perpendicular axis, *y*, had 300 mm of encoded linear travel, although this was not utilised in the research reported here. Camera height in the *z* axis was measured from the floor to the sensor plane of the camera. Reflective markers were for a VICON motion capture system used to calibrate the equipment. The target ‘tree’ projected a shadow on both sides as it was central to overhead environmental lighting, the effect of which is later illustrated in the responses shown in Fig. 11. The background and target remained stationary for all testing, with the camera being attached to the y-axis platform which itself traversed the x-axis

To fulfil these requirements, a custom 2 degree-of-freedom linear platform was constructed for this research, shown in Fig. 2. The longitudinal x-axis provided 2600 mm of encoded linear travel at velocities from $\sim$20 mm/s through to $\sim$130 mm/s. The perpendicular y-axis was capable of providing 300 mm of encoded linear travel at the same velocities as the x-axis. However, this functionality was not utilised for these experiments. This equipment is similar in concept to that used by Schwegmann et al. (2014). However, it is capable of recording in real-time at 100 frames per second and undertaking complex planar motion in 2 dimensions (*x* and *y*), noting the latter was not utilised in this specific research. While the camera can achieve higher frame rates, the frame rate was kept at 100 frames per second as: 1) It is a multiple of the local AC power frequency; and 2) the velocity profile of the experimental equipment was matched with the biological limitations of the algorithm assuming a 100 Hz sampling rate. The background and target remained stationary throughout all testing, with the camera being attached to the y-axis platform and this platform traversed the x-axis. This configuration produces a maximal motion energy magnitude when the object of interest is perpendicular with the observer. By disregarding the angular content of the optic flow vector, the magnitude can be considered to be the time to impact the object, regardless of whether the object is within the path of the observer or not, i.e. the time to impact if the direction of motion was directly towards the camera. This value alone (without the angular component) is potentially very useful as it serves as a relative estimation of distance to the object. Furthermore, in a situation where the observed object is assumed to be stationary, it can be converted to an absolute distance estimation with the fusion of kinematic information about the camera platform itself.

Computational power was provided by an NVIDIA Jetson TK1 and all processing was performed using only the CPU cores. Recordings were saved uncompressed in a custom binary file format, ensuring complete and faithful reproduction of the raw camera data without the added computational overhead of, or possible loss of data from, data compression algorithms.

Environmental lighting was controlled (e.g. no natural lighting) and featured overhead cool white ($\approx$4000 K) LED light fixtures. The stationary target was positioned directly under a light fixture, with equal spacing to the next fixtures on either side. This produced a relatively uniform shadow pattern behind the target.

The video sequences were captured in Polar format (due to the omnidirectional lens used). These were unwrapped at the maximum resolution possible without oversampling a pixel. An optical blur was then applied to mimic the acceptance angles of biology, and finally, the image was downscaled to the final working resolution of 180 horizontal pixels by 36 vertical pixels. This process followed that used for rotational motion and is detailed in-depth in (Skelton et al. 2019).

### Time to impact algorithm

**Fig. 3:**
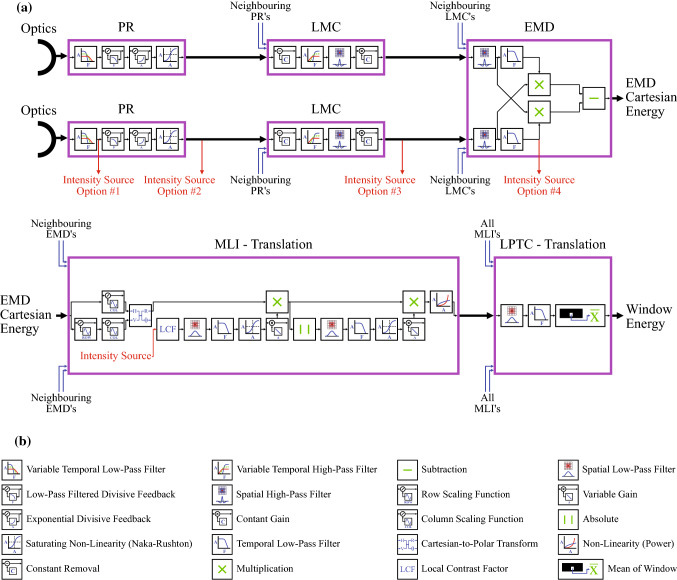
Diagrammatic representation of: **a** Our proposed algorithm, consisting of models of the photoreceptor (PR), lamina monopolar cells (LMC), elementary motion detector (EMD) modelled as a Hassenstein–Reichardt detector, what we call the medulla-lobula interneurons (MLI), and the lobula plate tangential cells (LPTC); and **b** the legend associated with this representation. On the PR, LMC, and EMD stages, there are 4 possible points where intensity can be fed forward into the MLI model for dynamic energy normalisation by contrast. See main text for justifications for these 4 options, and how the final option was selected. For best viewing, please see the online version of this article

Building on an existing rotational optic flow velocity estimation algorithm (Skelton et al. 2019), a novel algorithm was developed for estimating the time to impact during translational motion in a challenging contrast environment. The complete algorithm can be seen in Fig. 3. It is comprised of distinct and separable models of various stages of the visual pathways of insects.

#### Photoreceptor (PR) model

The photoreceptors (PR) are part of the retina region and are responsible for phototransduction (conversion of photons into electrical signals), dynamic range compression, and dynamic gamma correction, all to normalise the input luminance (Drews et al. 2020).

#### Lamina monopolar cells (LMC) model

The lamina monopolar cells (LMC), which reside in the lamina region of the brain, are responsible for signal conditioning. Their primary purposes are the removal of redundant information, in a spatio-temporally optimal way, and edge enhancement (van Hateren 1992).

#### Elementary motion detector (EMD) model

The elementary motion detectors (EMD), which are modelled as Hassenstein–Reichardt detectors (Hassenstein and Reichardt 1956) and shown in Fig. 3a, are responsible for estimating the motion energy. Since the model was applied to rectangular representations of the images, optic flow was calculated against both the nearest neighbours (cardinal directions), and the next-nearest neighbours (ordinal directions).

Our model of the EMD includes additional spatial high-pass filtering prior to the temporal low-pass filter on the delay arm of the HR-EMD. While the output of the preceding stage (LMC) ends with a linear transform following the spatial high-pass filter there, separable spatial high-pass filters have been used to represent different biological processes. These include small target detection, which requires a different signal characteristic out of the LMC when compared to the egomotion estimation processes (Melville-Smith et al. 2019). As such, to make this model for translational optic flow estimation compatible with related models for target detection, separable high-pass filters are retained.

Furthermore, some models of the LMC contain a compressive nonlinearity following the spatio-temporal filtering (Brinkworth and O’Carroll 2009). To remain compatible with such models, separate filtering at the start of the EMD stage is required, since it cannot be amalgamated with the LMC filtering.

#### Medulla-lobula interneuron (MLI) model

The medulla-lobula interneuron (MLI) is a generic term for processing thought to occur between the EMD stage, which has strong neurophysiological support (Hassenstein 1951; Hassenstein and Reichardt 1956; Reichardt 1962; Haag et al. 2004), and the lobula plate tangential cells (LPTC’s) that have strong and growing neurophysiological support (Borst et al. 2010; Hardcastle and Krapp 2016; Longden et al. 2017).

In this research, the MLI serves 4 distinct functions. First, the Cartesian energy estimates produced by the EMD model—which are estimates of the horizontal and vertical motion present within the scene—require scaling to account for the distortion to the optic flow introduced by the panoramic lens. Next, dynamic nonlinear contrast adaptation occurs where areas of low contrast are gained and areas of high contrast attenuated. Dynamic nonlinear motion adaptation then follows, where areas of low motion are gained and areas of high motion attenuated. Finally, a Naka-Rushton nonlinearity adjustment is performed to redistribute the motion energy estimates around a desired operating point. For example, a skewed distribution of motion energy—something that is entirely possible with the preceding nonlinear adaptations—can be transformed to a more normal distribution (Severns and Johnson 1993). This is a critical transformation that is required by follow-on mathematical functions that have specific ranges in which they are expecting the signal to be, and mimics biological neurons that have defined operational bandwidths.

Optical Scaling: To determine the correct scaling and directionality of the optic flow estimations, the *de facto* method comes from Koenderink and van Doorn (1987) which aims to accurately estimate the per-pixel optic flow vector, especially when using fish-eye, wide-angle, or omnidirectional lenses (Schwegmann et al. 2014). However, the small optical resolution of our imagery, intentionally set to the ommatidial resolution of 180 pixels to represent 360$^\circ$ horizontal field of view (FOV) and 36 pixels to represent 53$^\circ$ vertical FOV—a resolution shown to be optimal for optic flow under rotational motion (Brinkworth and O’Carroll 2009)—causes singularities around the point of expansion and point of contraction. Additionally, assuming perfect methodological set-up, the cardinal directions fall between 2 columns of pixels. To overcome this, we implemented custom scaling functions on the horizontal and vertical optic flow fields, with an arbitrary offset away from 0. First, the horizontal scaling factor, $\alpha$, is only dependent upon the current column:

6 $$\begin{aligned} \begin{aligned} \alpha \left( i_c\right) = {}&S_{h}\left( i_c\right) \Bigg (C + \left( 1 - C \right) *\Bigg . \\&\Bigg . {-\cos } \left( 2 \pi \frac{ i_c - \left\lfloor i_c *N_q / N_c \right\rfloor }{ \left( N_c - N_q \right) } \right) \Bigg ) \end{aligned} \end{aligned}$$

**Fig. 4:**
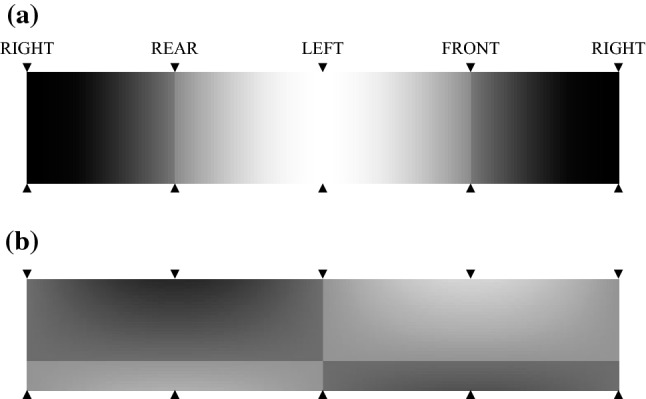
Scaling gradients applied to the output of the elementary motion detector (EMD) model at the start of the medulla-lobular interneuron (MLI) model. Gradients exist in $[-1.0, 1.0]$ in application, denoting negative and positive optic flow energies, respectively. However, these have been rescaled to [0.0/black, 1.0/white] for display purposes. **a** The horizontal scaling component, where black denotes perpendicular right, white denotes perpendicular left, and the discontinuities at front and rear of image represent a flat offset to deal with the point of expansion and point of contraction singularities. **b** The vertical scaling component, where tending towards black denotes maximal optic flow away from camera and tending towards white denotes maximal optic flow towards camera. The horizontal discontinuity (row 25) represents the horizon of the camera field of view (FOV), and the vertical discontinuities at left and right indicate the perpendiculars where no vertical optic flow occurs. These compensate for the point of expansion and point of contraction singularities. Unlike the horizontal scaling (FOV = 360$^\circ$), the vertical scaling (FOV = 53$^\circ$) does not reach the extremes of the $[-1.0, 1.0]$ range

Where $S_{h}\left( i_c\right)$ is a horizontal *signum* scalar used to produce the correct directionality around the discontinuities, *C* is a flat offset to deal with point of expansion and point of contraction, $i_c$ is the current column index, $N_q$ is the number of sections across the horizontal FOV (4 for front, right, back, and left), and $N_c$ is the number of columns in the image. The value of *C* = 0.15 was chosen empirically as it provided no perceptible motion artefacts around the point of expansion or point of contraction. Using (6), $\alpha$ exists within a discontinuous range of $\left[ -1.0, -C \right]$ and $\left[ C, 1.0 \right]$. The resulting gradient can be seen in Fig. 4a, where the discontinuities caused by the arbitrary offset can be seen at 1/4 (REAR) and 3/4 (FRONT) of image width.

The vertical scaling factor has both a column dependence and a row dependence. The vertical column component, $\beta _{c}$, is similar to the horizontal scaling factor:

7 $$\begin{aligned} \beta _{c}\left( i_c\right) = -\sin \left( 2 \pi \frac{ i_c - \left\lfloor i_c *N_q / N_c \right\rfloor }{ \left( N_c - N_q \right) } \right) \end{aligned}$$

Where as the vertical row component, $\beta _{r}$, is:

8 $$\begin{aligned} \beta _{r}\left( i_r\right) = \sin \left( \theta _{v,p} - i_r \frac{\left( \theta _{v,p} - \theta _{v,n} \right) }{N_r - 1} \right) \end{aligned}$$

where $\theta _{v,p}$ is the vertical positive FOV, $i_r$ is the index of the current row, $\theta _{v,n}$ is the vertical negative FOV, and $N_r$ is the number of rows in the image.

The final vertical scaling factor, $\beta$, is:

9 $$\begin{aligned} \beta = S_{h} *S_{v} *\left( C + \left( 1 - C \right) *\beta _{r} *\beta _{c} \right) \end{aligned}$$

where $S_{v}$ is a vertical *signum* scalar used to produce the correct directionality around the discontinuities. These values are entirely dependent upon the exact methodological set-up used in this research, such as the orientation of the camera and the preferred directionality, and are trivially derivable based on the 4 quadrants of the vertical scaling gradient. The vertical scaling factor also exists within a discontinuous range of $\left[ -1.0, -C \right]$ and $\left[ C, 1.0 \right]$. However, as the vertical FOV does not span the full 360$^\circ$, the absolute upper limits of these ranges are not reached. Figure 4b shows the horizontal discontinuity around the horizon (approximately row 25) and the vertical discontinuities around the RIGHT and LEFT perpendiculars.

After this point, we change to dealing with the energy in Polar coordinates with magnitude *R* and direction $\theta$. One major benefit of working in Polar coordinates is that the EMD suffers from the *aperture problem* (Reichardt et al. 1988). This presents as the inability to accurately estimate the true direction of optic flow in a local neighbourhood, which causes instability in the direction output $\theta$, although the magnitude *R* would be unperturbed. This presents as the inability to accurately estimate the true direction of optic flow in a local neighbourhood. This presents as an instability in the direction output $\theta$, although the magnitude *R* would be unperturbed.

Contrast Adaptation: Contrast adaptation in biology has been shown to be a temporal process (Matulis et al. 2020). It is also thought to be driven by separable ON–OFF neural pathways (Drews et al. 2020), where different neuronal processing occurs based on the contrast transition from dark-to-light (ON) and light-to-dark (OFF). As the algorithm proposed in this research does not extend its already extensive elaborations of the complete neural pathway to include separate ON–OFF pathways, the integration of that theory into practice is somewhat limited. The fundamental principle behind how we approach contrast adaptation is based on spatio-temporal processes used previously for motion adaptation (Brinkworth and O’Carroll 2009; Skelton et al. 2019).

First, the contrast of the image is calculated to generate a contrast map. However, the exact input image used, or the intensity source, for generating this contrast map is unknown. While it is unclear from literature whether contrast adaptation is a feedback or feedforward process (Drews et al. 2020), the contrast information is largely lost once the optic flow estimation occurs. Therefore, four possible locations were identified for feedforward contrast adaptation within this specific algorithm, as shown in Fig. 3: (1) the temporal adaptation level of the PR (essentially a history of the luminance input); (2) the output of the PR prior to entry into the LMC; (3) the output of the LMC before entry into the EMD; and (4) the delay arm of the Hassenstein–Reichardt detector within the EMD’s. Option 4 was chosen based on results presented later in Sect. 4.1.

The elaborated Hassenstein–Reichardt detector weights the optic flow in the cardinal and ordinal directions base on the geometric distances (1 and $1 / \sqrt{2}$, respectively) (Hassenstein 1951; Hassenstein and Reichardt 1956). Therefore, a typical measure of local contrast, such as the root mean square (RMS) contrast, is not suitable (Peli 1990). To account for scenes with differing luminance ranges, the contrast should also be weighted by some factor of the global luminance. In this work, a previously published metric of local contrast, known as a local contrast factor (LCF), was used (Skelton et al. 2019). This metric was built upon the work of Matkovic et al. (2005) by incorporating weighting for cardinal and ordinal directions. It begins by calculating the absolute pixel difference in the local neighbourhood:

10 $$\begin{aligned} \begin{aligned} C_{nn} = {}&\vert I_{x,y} - I_{x,y-1} \vert + \vert I_{x,y} - I_{x-1,y}\vert \\&+ \vert I_{x,y} - I_{x+1,y}\vert + \vert I_{x,y} - I_{x,y+1}\vert \end{aligned} \end{aligned}$$

11 $$\begin{aligned} \begin{aligned} C_{nnn} = {}&\vert I_{x,y} - I_{x-1,y-1} \vert + \vert I_{x,y} - I_{x+1,y-1}\vert \\&+ \vert I_{x,y} - I_{x-1,y+1}\vert + \vert I_{x,y} - I_{x+1,y+1}\vert \end{aligned} \end{aligned}$$

where *c* *nn* is the contrast of pixel at *x*, *y* relative to the nearest neighbours (cardinal directions), $C_{nnn}$ is the contrast of pixel at *x*, *y* relative to the next nearest neighbours (ordinal directions), and *I* is the pixel intensity for a pixel at coordinate locations *x*, *y*. These specific equations relate to interior ‘fill’ pixels. The ‘edge’ and ‘corner’ pixels are processed in the same manner with the relevant terms for pixels that do not exist dropped, such as *x*-1 pixels for an *x*=0 pixel, or *y*-1 pixels for a *y*=0 pixel. The local contrast for a pixel at *x*, *y* is then calculated as:

12 $$\begin{aligned} C_{x,y} = \frac{\alpha *C_{nn} + \beta *C_{nnn}}{\alpha *N_{nn} + \beta *N_{nnn}} *1 \Big / \overline{\vert I \vert } \end{aligned}$$

where $\alpha$ = 1 is the weighting factor for the nearest neighbours, $\beta$ = $1/\sqrt{2}$ is the weighting factor for the next nearest neighbours, $N_{nn}$ is the number of nearest neighbours (4 for ‘fill’, 3 for ‘edge’, and 2 for ‘corner’), $N_{nnn}$ is the number of next nearest neighbours (4 for ‘fill’, 2 for ‘edge’, and 1 for ‘corner’), and $\overline{\vert I \vert }$ is the mean of the absolute intensity.

Having calculated the per-pixel contrast (contrast map), a spatio-temporal blur is applied. The spatial filter is realised as a Gaussian low-pass filter, where the kernel size is derived from a specified full-width at half maximum (FWHM) of the Gaussian distribution of the filter, $\sigma _G$. The temporal filter is realised as a temporal low-pass filter, where the cut-off frequency, $f_c$, is fixed across the entire image. Next, a Naka-Rushton transform is used to provide nonlinear weighting to different segments of the contrast map. This nonlinear gain map is then applied to the magnitude of the optic flow after optical corrections have been applied.

Motion Adaptation: The motion adaptation used in this work is the same fundamental procedure as used in (Brinkworth and O’Carroll 2009; Skelton et al. 2019). Much like the generation of the contrast map used previously, the absolute magnitude of the motion energy estimate is spatio-temporally blurred to generate a motion adaptation map. This is then transformed using a Naka-Rushton transform to construct a nonlinear gain that is applied to the input motion energy.

Nonlinearity Adjustment: The final function of the MLI is to perform a nonlinearity adjustment to the contrast and motion adapted signals. Historically, this was implemented as a square-root operation to correct the squaring that occurs within the EMD’s (Brinkworth and O’Carroll 2009). However, it was found in later work (Skelton et al. 2019) that the algorithm exhibits stronger performance when this nonlinearity adjustment uses a gain factor $k>1$, making the signal more nonlinear.

#### Lobula plate tangential cells (LPTC) model

Part of the lobula plate tangential cells (LPTC) are responsible for encoding the motion energy estimates into signals, be that graded (HS) or spiking (H1), for the motor neuron control centres to influence the flight behaviour of the insect (Haag and Borst 1997; Longden et al. 2017). In this research, the HS cells were realised as a spatio-temporal pooling function, encoded as a spatial low-pass filter followed by a temporal low-pass filter. From this spatially pooled representation of the adapted optic flow estimates, a region of interest—known as a receptive field in biology—was taken from the left quadrant of the image. Specifically, the mean of a window 2 pixels wide and 8 pixels high centred around the horizon and the left extreme was used. This window was perpendicular to the direction of motion of the camera, and this experienced the maximum optic flow as the camera translated past a target.

### Comparative models

To show the improvements of the algorithm proposed in this research, it was statistically compared to biologically inspired algorithms featuring lower levels of elaboration, thus allowing for quantification of the improvement of each elaboration.

The most basic algorithm was formed using a non-elaborated (no additional spatial high-pass filtering) Hassenstein–Reichardt elementary motion detector (HR-EMD). This estimates motion energy across cardinal and ordinal neighbours within a local 3x3 pixel neighbourhood. To emulate a receptive field, the LPTC-translation model proposed in this research, which is a spatio-temporal filter and spatial pooling function, is also used. This was used as the baseline for comparative purposes.

Secondly, the elaborated pre-processing stages, namely the photoreceptor (PR) and lamina monopolar cells (LMC), as well as the additional pre-HR-EMD spatial high-pass filtering, was used with the LPTC-translation model. This is functionally the fully elaborated algorithm proposed with the novel contrast adaptation and motion adaptation processes that are present in the medulla-lobula interneurons (MLI) disabled. This version represented a translational equivalent to previously published work (Brinkworth and O’Carroll 2007).

Thirdly, the algorithm proposed in this research is used with the novel approach to contrast adaptation within the MLI model disabled. This configuration is strikingly similar to a previously published rotational velocity estimation model (Skelton et al. 2019), where the major difference is the lack of an adaptive noise suppression process that was present within the LPTC model that was instrumental in facilitating environment invariance for that application. The spatio-temporal filtering that occurs within the receptive field LPTC model of the proposed algorithm acts like a noise suppression filter.

Finally, the fully elaborated algorithm proposed in this research, including the nonlinear spatio-temporal feedforward filtering for contrast invariance, rounded out the testing configurations.

**Table 2:**
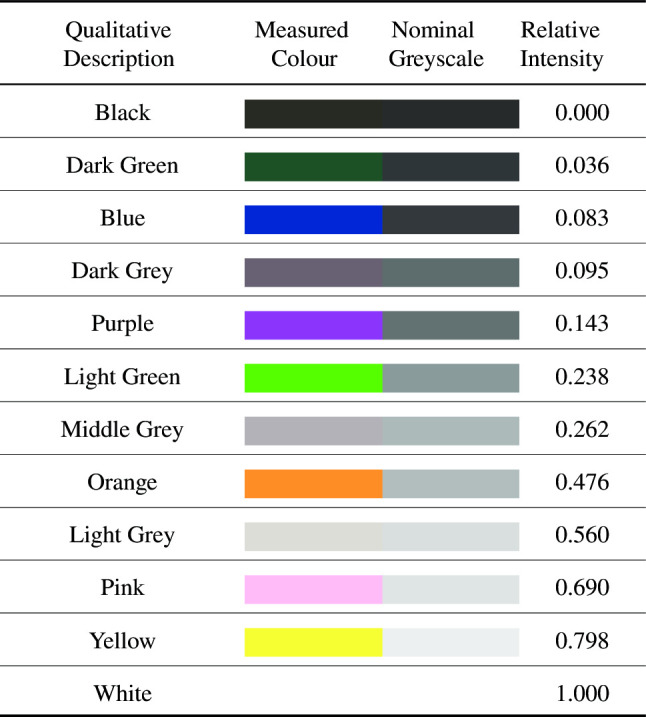
Different colours of backgrounds used in this research with a qualitative description (the visual colour referred to for simplicity), the nominal colour in RGB (captured using a Samsung Galaxy S7 with all colours in frame under identical lighting conditions), the nominal RGB colour converted to greyscale using (13), and the relative intensity measured by the monochromatic system used in this research.

Differences between the perceptual gradient of the nominal greyscale and the numerical gradient of the relative intensity were likely caused by different lighting conditions perceived by the 2 distinct camera systems

### Dataset

For this research, a comprehensive calibration dataset was captured. Common fabric was used as a means to obtain different colours of background that presented to our monochromatic system as different shades of grey. These different backgrounds were utilised as a controlled method of testing the contrast dependence of the estimation of the time to impact. Using the recommendations widely used from Rec. ITU-R BT.601-7, a representative greyscale value can be obtained from a nominal RGB values using:

13 $$\begin{aligned} Y_{x,y} = 0.2989R_{x,y} + 0.5870G_{x,y} + 0.1140B_{x,y} \end{aligned}$$

where subscript *x*, *y* denotes a pixel coordinate, *Y* is the calculated greyscale, or luminance, value for the output pixel, *R* is the red component of the input pixel, *G* is the green component of the input pixel, and *B* is the blue component of the input pixel. The descriptive colour of the different fabrics, the nominal RGB colour, the nominal greyscale value, and the relative intensity of each colour with respect to the dataset can be seen in Table 2, where black has been normalised to 0.0 and white normalised to 1.0.

**Fig. 5:**
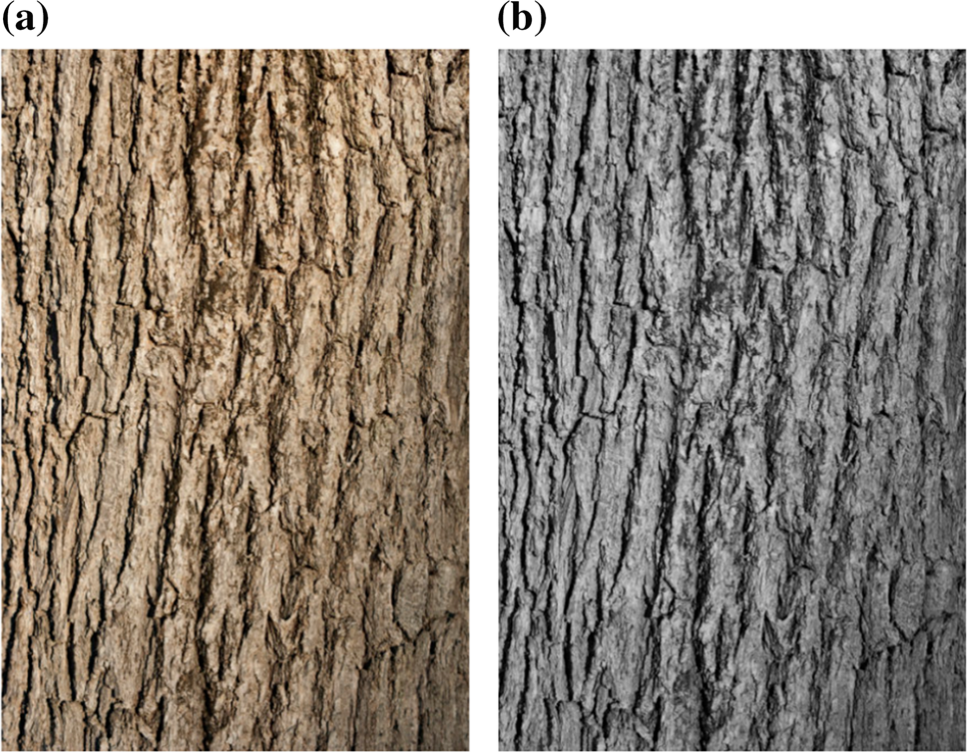
Flat ‘skin’ of a tree used to wrap a 90 mm PVC pipe to create a tree analogue with uniform diameter. Image was printed at 287 mm wide and 400 mm high (A3 paper with printer borders). This tree texture closely resembles that of native trees in the vicinity of the laboratory. **a** Original colour skin. **b** Converted to greyscale as both our camera system and our computer vision algorithm are monochromatic. Image (**a**) from www.freeimages.co.uk with (**b**) derived from (**a**)

To construct the targets, 90 mm PVC pipe was used as it is readily available, inexpensive, and dimensionally accurate between batches. Sections of 1200 mm length were used. A tree skin (Fig. 5a) was converted to monochrome (Fig. 5b) and printed at a physical size of 287 mm *x* 400 mm (A3 paper with printer borders) and glued to the PVC pipe to form a tree analogue. Although 3 individual skins were required to cover the entire 1200 mm length of the target, simple mirroring of the texture allowed for alignment between edges. Due to the fractal nature of a pattern such as a tree, the seams and the resulting repeating pattern were barely distinguishable to a human observer.

The camera rail was positioned at different distances from the target. From Sect. 3.1, the minimum distance, $L_\text {min}$, was 200 mm, while maximum distance, $L_\text {max},$ was 1000 mm. The Hassenstein–Reichardt elementary motion detector (EMD) model requires the motion per frame to be less than 0.5 pixels, or aliasing will occur. This is a side effect of the digital model of the analogue biological system combined with the frame rate that a technological implementation operates at compared to the analogue processing that occurs in nature. Based on the requirement of 0.5 pixels of motion per frame, the maximum achievable velocity at a given distance to target, $v_\text {max}$ can be approximated using:

14 $$\begin{aligned} v_\text {max} = f_s L \tan \left( \frac{\theta _p}{2} \right) \end{aligned}$$

where $f_s$ is the sampling frequency (frame rate), *L* is the actual distance to target, and $\theta _p$ is the angular resolution per pixel which was 2$^\circ$ for the ommatidial resolution of 180 pixels wide that our algorithm is designed to work with. For the experimental set-up of this research, at a distance of $L_\text {min}$ = 200 mm, $v_\text {max}^{200}$ = 349.101 mm/s, and this increases as the distance to target increases. This style of algorithm can be tuned to have a soft limit or maximum response below this $v_\text {max}$ value, such as has been shown previously in (Brinkworth and O’Carroll 2009; Skelton et al. 2017, 2019).

The resulting combinations of distances and velocities, and the corresponding time to impact recorded for each distance, can be seen in Table 3. A tick mark ($\checkmark$) indicates that every frame of the recording was used. A cross mark ($\times$) indicates that every other frame was used to increase the perceived velocity of the system. Where both a tick mark and a cross mark ($\checkmark$ $\times$) are present, this indicates the recording was used both with and without decimation. For example, a distance of 400 mm at 85.32 mm/s would also be decimated to a velocity of 170.64 mm/s. This decimation allowed for processing of velocities that exceeded the capabilities of the experimental platform, while still respecting the limitations of the algorithm. Finally, a dash (–) indicates that no recording or decimations occurred at this combination.

**Table 3:** Velocity and distance pairs that make up the recordings

	Distance to Target (mm)					
	200	300	400	600	800	1000
Velocity (mm / s)						
21.33	$\checkmark$	–	–	–	–	–
32.00	$\checkmark$	$\checkmark$	–	–	–	–
42.66	$\checkmark$	–	$\checkmark$	–	–	–
63.99	$\checkmark$	$\checkmark$	$\checkmark$	$\checkmark$	–	–
85.32	$\checkmark$	–	$\checkmark$ $\times$	–	$\checkmark$ $\times$	–
95.99	–	$\checkmark$ $\times$	–	$\checkmark$ $\times$	–	–
106.65	$\checkmark$	–	$\times$	–	–	$\checkmark$ $\times$
127.98	$\checkmark$	$\checkmark$	$\checkmark$ $\times$	$\checkmark$ $\times$	$\checkmark$ $\times$	–
Time to Impact (s)						
1.563	$\checkmark$	$\times$	$\times$	–	–	–
1.875	$\checkmark$	–	$\times$	–	–	–
2.344	$\checkmark$	$\checkmark$	$\times$	$\times$	–	–
3.125	$\checkmark$	$\checkmark$	$\checkmark$	$\times$	$\times$	–
4.688	$\checkmark$	$\checkmark$	$\checkmark$	$\checkmark$	$\times$	$\times$
6.251	$\checkmark$	–	$\checkmark$	$\checkmark$	$\checkmark$	–
9.376	$\checkmark$	$\checkmark$	$\checkmark$	$\checkmark$	$\checkmark$	$\checkmark$

Pairs were selected to ensure multiple distances were present at each resulting time to impact. A tick mark ($\checkmark$) indicates that the velocity–distance pair was used as is. A cross mark ($\times$) indicates that every other frame was decimated during playback to increase the velocity of the recording. Where both marks ($\checkmark$ $\times$) are present, the recording was used both with and without decimation. For example, a distance of 400 mm at 85.32 mm/s ($t_I$ = 4.688 s) would also be decimated to a velocity of 170.64 mm/s ($t_I$ = 2.344 s). This decimation allowed for processing of velocities that exceeded the capabilities of the experimental platform, while still respecting the limitations of the algorithm

Several factors were important when determining these combinations. Firstly, due to the increased texture present in the target at 200 mm distance, this distance was required at all times to impact. Secondly, at least 2 different distances are required at each time to impact. Thirdly, due to concerns about introducing flickering from the artificial lighting in the environment, no more than every other frame could be skipped, effectively doubling the achievable recording velocity.

For the 360 different combinations of background colours, distances to target, and camera velocities, the dataset contains 779,267 frames. However, due to the same spatial sample being recorded for different velocities—that is, the camera commenced and terminated recording at the same position on the rail for each recording, irrespective of velocity—only 321,565 frames were used for analyses after the recordings at the slower velocities were clipped.

### Time to impact calculation

Using the ground-truth distance to target from the experiment, combined with the known velocity of the experimental platform (encoded drive motor), the ground-truth time to impact was calculated as:

15 $$\begin{aligned} t_{I} \left[ s\right] = \frac{L}{v} \left[ \frac{\text {mm}}{\text {mm} / \text {s}}\right] \end{aligned}$$

where $t_{I}$ represents the time to impact the target in seconds, *L* denotes the linear distance to the target in millimetres, and *v* is the linear velocity of the experimental platform in millimetres per second. The linear distance to target, *L*, was taken as the distance between the optical centre of the camera lens and the physical centroid of the target. This was done as the ommatidial resolution of our system ($180\times 36$) renders the naturalistic texture within the target undetectable at higher distances. At the lower values of L, such as 200 mm, the texture within the object that is the most prevalent source of optical energy is at 155 mm, not 200 mm. However, at the larger values of *L*, such as 1000 mm, the resultant distance between face and optical centre is now 945 mm; a much lower difference. This error was deemed to be acceptable as, in a real-world situation, it is most likely that an arbitrarily large time to impact would be used as a ’buffer’ around targets.

### Estimation of time to impact

The algorithm proposed in this work does not directly measure the time to impact. This is because the available degrees of sensing are less than the number degrees of freedom for the system. Unlike rotational motion, where the motion energy is directly related only to the rotational velocity, translational motion has two degrees of freedom: (1) the distance between the observer (e.g. camera) and the object; and (2) the relative velocity between those two. Without having *a priori* knowledge of one of these pieces of information (e.g. an accurate map of the environment), it is not possible to directly compute the time to impact from the motion energy. While the platform odometry could certainly be used to remove one of these degrees of freedom and compute a time to impact directly, this work focussed on the more general case where this information may not be available and, hence, must estimate the time to impact based on algorithm calibration.

Instead, it estimates the motion energy in a given direction. To convert this motion energy into a usable time to impact estimate, previous rotational velocity work (Skelton et al. 2017) has used a logistic curve as it is known that this style of highly elaborated, biologically inspired optic flow algorithm produces a sigmoidal response curve on a log-lin graph:

16 $$\begin{aligned} E_m = \frac{L}{1 + \exp {\Big (-k\big (\ln \left( v_\text {est}\right) - v_\text {mid}\big )\Big )}} \end{aligned}$$

where $E_m$ is the motion energy estimate from the model, *L* is the maximum expected curve value, *k* is the steepness of the curve, $v_\text {est}$ is the velocity estimate, and $v_\text {mid}$ is the midpoint of the sigmoid. To produce a velocity estimate, this is rearranged:

17 $$\begin{aligned} v_\text {est} = \exp {\left( \frac{\ln \left( \frac{L}{E_m} - 1\right) }{-k} + v_\text {mid}\right) } \end{aligned}$$

However, it was expected that, due to the inverse-square law, the response of the algorithm presented in this research would require a more complex transform. Therefore, this research used the generalised form of the logistic curve:

18 $$\begin{aligned} E_m = A + \frac{K - A}{\Big (C + Q *\exp {\big ( -B *\ln \left( t\right) \big )\Big )} ^ {1 / V}} \end{aligned}$$

where *A* is the lower asymptote, *K* is the carrying capacity, *C* relates to the upper asymptote and is typically 1.0, *Q* relates to the value of $E_m\left( 0\right)$, *B* is the growth rate, and *t* is the time to impact. Rearranging this to produce a time to impact estimate based on the motion energy output of the algorithm:

19 $$\begin{aligned} t = \exp \left( \frac{\ln \left( \left( \left( \frac{K - A}{E_m - A}\right) ^{V} - C \right) \Big / Q\right) }{-B}\right) \end{aligned}$$

This estimate of the time to impact, in seconds, will later be fit to the experimental data obtained, as shown in Sect. 4.2.

### Quantification of accuracy

To statistically quantify the ability of our algorithm to discriminate between each time to impact, we adapted a previously published metric that quantifies the ability of an algorithm to statistically discriminate between optic flow outputs across different scene and velocity pairs, known as an adjusted geometric score, or $G_{\text {adj}}$ (Skelton et al. 2019). This metric has historically been used to quantify data where the algorithm response is increasing as a function of the control variable, the sensor velocity. This paper deals with a decreasing response for the algorithm as the time to impact increases. Consequently, minor rearrangement of the metric was required. First, the distinctiveness of a response is calculated by:

20 $$\begin{aligned} \begin{aligned} D_i = {}&{{\,\textrm{PPD}\,}}_i *\Bigg ( \frac{ \big ( P50_{i - 1} - P50_{i} \big ) }{ \big ( P50_{i - 1} - P5_{i - 1} \big ) + \big ( P95_{i} - P50_{i} \big ) } \Bigg ) \end{aligned} \end{aligned}$$

where *i* and $i-1$ are the indices of the current and previous rotational velocity, respectively, *D* is the distinctiveness score, and *P*5, *P*50, and *P*95 are the 5th, 50th, and 95th percentiles, respectively. The goal is to maximise $D_i$. The ${{\,\textrm{PPD}\,}}_i$ term is the number of test points per decade, and was calculated using:

21 $$\begin{aligned} {{\,\textrm{PPD}\,}}_i = \frac{ 1 }{ \log _{10} \big ( {t_I}_{i} \big ) - \log _{10} \big ( {t_I}_{i - 1} \big ) } \end{aligned}$$

where ${t_I}_{i}$ is the time to impact at index *i*, and ${t_I}_{i - 1}$ is the time to impact at index *i*-1. This correction was required to account for the logarithmically spaced time to impacts. To account for any non-Gaussian distribution of the dataset, the raw geometric mean of *D*, ${\bar{G}}_{\text {raw}}$, was calculated across the entire dataset by means of a natural logarithm transform:

22 $$\begin{aligned} {\bar{G}}_{\text {raw}} = \exp \bigg (\frac{1}{N_v - 1}\sum _{i=2}^{N_v}\Big (\ln \big (D_i\big )\Big )\bigg ) \end{aligned}$$

where $N_v$ is the number of test velocities. ${\bar{G}}_{\text {raw}}$ is thus a measure of how the statistical distribution of motion energy responses differs between test velocities. The geometric confidence intervals were also calculated using a natural logarithm transform and are used to demonstrate the variability of ${\bar{G}}_{\text {raw}}$ throughout the range of tested velocities. The final form of the metric, the adjusted geometric score $G_{\text {adj}}$, accounts for the confidence range:

23 $$\begin{aligned} G_{\text {adj}} = {\bar{G}}_{\text {raw}} - \big (G_U - G_L\big ) \end{aligned}$$

where $G_U$ and $G_L$ are the upper (95%) and lower (5%) geometric confidence intervals, respectively. The $G_{\text {adj}}$ is a measure of not only how well an algorithm can discriminate between test velocities, as calculated by ${\bar{G}}_{\text {raw}}$, but also the consistency of those discriminations across the velocity range sampled. It is scale and unit invariant and thus requires no normalisation or standardisation of algorithm outputs. This allows for direct statistical comparison between numerous algorithms without the requirement of having ground-truth optic flow measurements.

### Quantification of intensity rank ordering

It is well known that correlation-based algorithms, such as the HR-EMD, are inherently contrast-dependent. To quantify this dependence, the Spearman’s rank correlation coefficient (Hinkle et al. 2003), $\rho$, was used as it is a well-known statistical measure of the correlation between the rank ordering of two variables:

24 $$\begin{aligned} r_s = 1 - \frac{6 \sum \limits _{i=1}^{n} d_i^2}{n(n^2 - 1)} \end{aligned}$$

where $r_s$ is the sample statistic, $d_i$ is the difference in ranks between sample *X* and *Y*, and *n* is the number of samples. In this work, the two samples were: *X*, the expected rank based on the ordered intensities as presented in Table 2; and *Y*, the ranked LPTC magnitude, e.g. as presented in Fig. 10.

While the rank ordering of a single sample set, such as a given background, distance to target, and time to impact combination, could be of some use, this work is more interested in the generalised performance across a wide range of experimental configurations. To this end, we will report the mean ± 95% CI of the Spearman’s correlation coefficient across all samples, ${\bar{\rho }}$, as calculated by:

25 $$\begin{aligned} {\bar{\rho }} = \frac{\sum \limits _{i=1}^{m} r_{s_i}}{m} \end{aligned}$$

where *i* is an experimental configuration (e.g. one of those shown in Table 3), *m* is the total number of experimental configurations, and $r_{s_i}$ is the sample statistic for that configuration, calculated using Equation (24).

To interpret the meaning of $\rho$, the rules of thumb from literature (Hinkle et al. 2003; Schober et al. 2018) will be used. Those are, for positive correlations: $0.0\le \rho \le 0.3$ = negligible; $0.3<\rho \le 0.5$ = low; $0.5<\rho \le 0.7$ = moderate; $0.7<\rho \le 0.9$ = high; $0.9<\rho \le 1.0$ = very high. The negative values hold true for negative correlations. These are, as stated in literature, arbitrary categorisations used to provide some form of basic guidance only.

The Python SciPy implementation of Spearman’s algorithm was used. The rank ordering, be that ascending or descending, bares no impact on the result as the correlation coefficient, $\rho$, is defined as $\rho \in {\mathbb {R}}~[-1.0,~1.0]$. However, as it is expected that a HR-EMD will produce ascending energy magnitudes as the contrast increases, and we can simplify the assumption that as background intensity increases, the contrast between background and target also increases, this work will use ascending rank where black $=~1$, dark green $=~2$, ..., yellow $=~11$, and white $=~12$.

### Algorithm tuning

When dealing with complex computer vision algorithms that have a growing number of parameters, a problem arises when optimally setting the parameter set of the algorithm. In previous work (Skelton et al. 2020), we presented a novel application of adaptive evolutionary algorithms for the purposes of assisting in the development of an elaborated rotational velocity estimation algorithm (Skelton et al. 2019). The reader is directed to that manuscript for full implementation details surrounding the adaptive genetic algorithm. Alternatively, the corresponding author of this manuscript can be contacted for further details.

For the research presented in this paper, tuning of the extensive parameter sets was again required. However, the complexity and extensiveness of this tuning is outside the scope of this paper, and will be reported in-depth elsewhere. In summary, an evolutionary algorithm was used to tune the parameter set of each algorithm outlined in Sect. 3.3 to a point of Pareto-optimality. Due to the complex nonlinear and symbiotic interactions present throughout the constituent models within the algorithm proposed, removing an individual or group of mathematical functions—for example the proposed novel contrast adaptation within the medulla-lobula interneuron (MLI)—may unfairly misrepresent the capabilities of the adjusted algorithm. Therefore, tuning the parameter set of each individual algorithm overcomes this bias and provides a level playing field for all algorithms.

### Software libraries

The C++ API of the OpenCV computer vision library, specifically version 4.1.0, was used for the implementation of the computer vision algorithms.

## Results

In this research, 4 different algorithms of varying progressions of biological elaboration have been used to provide context (see Sect. 3.3). This section describes the performance of each algorithm, and then closely analyses the characteristic behaviour of the proposed algorithm.

### Intensity source for contrast map

**Fig. 6:**
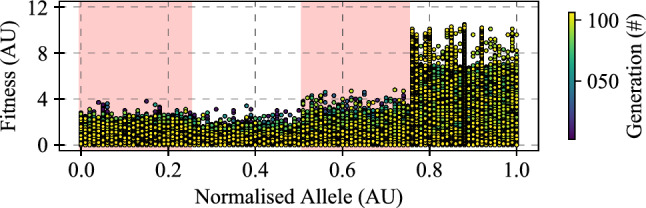
Tuning results for the intensity source for the novel contrast adaptation proposed within the medulla-lobula interneuron (MLI) model. Fitness refers to the statistical score calculated using (23) for different executions of the algorithm with different parameter sets. Option 1 exists for allele values of 0.0 to 0.25; Option 2 from 0.26 to 0.50; Option 3 from 0.51 to 0.75; and Option 4 from 0.76 to 1.0. Multiple alleles exist per option to aid integration into our existing evolutionary computation framework, and variability demonstrated within each option is caused by other parameters. All options are diagrammatically shown in Fig. 3. The stronger fitness values in response to Option 4 demonstrate the improvements possible by taking the intensity signals from this section of the elementary motion detector model to feedforward into the MLI model to use for dynamic contrast adaptation

There were 4 possible sources of intensity used to calculate the contrast map that was used for the novel contrast adaptation in the medulla-lobula interneuron (MLI) model (see Sect. 3.2.4). As part of the extensive tuning undertaken to facilitate this work (see Sect. 3.9), the full algorithm (previously referred to as Algorithm #4) was free to determine which intensity source resulted in the highest fitness output. The results of this search are shown in Fig. 6, where a very clear preference can be seen for Option 4, which was taken from the delay arm of the EMD model.

### Comparative algorithm statistical performance

**Fig. 7:**
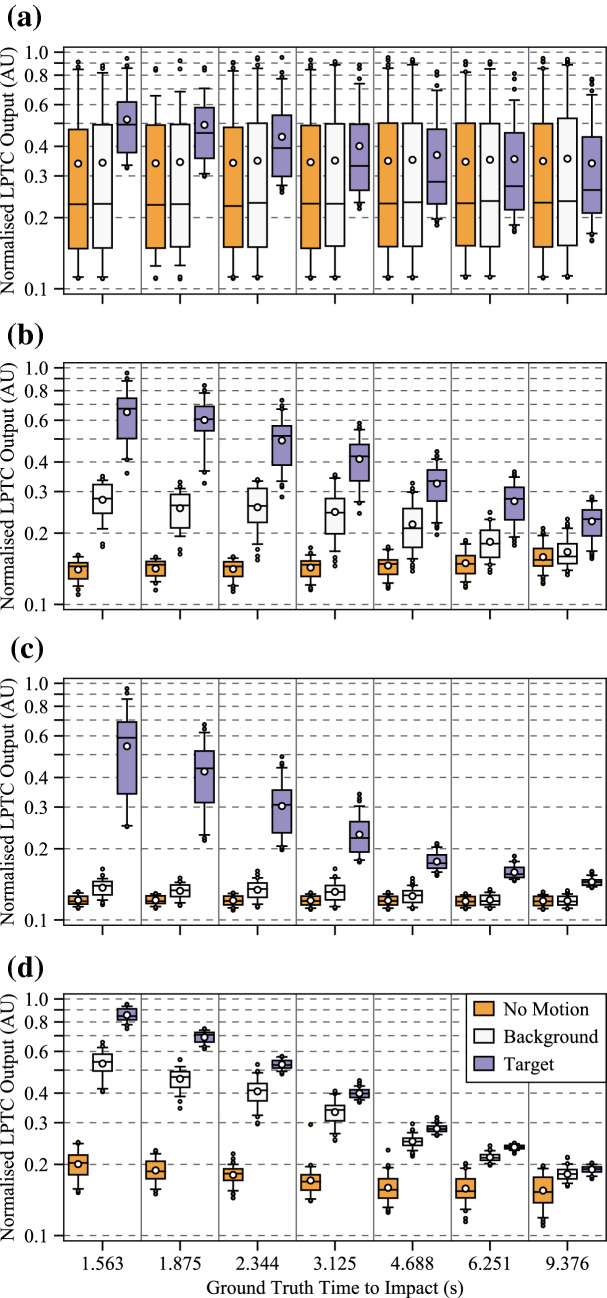
Algorithm response graphs showing energy estimates for no motion (left series, orange), the background (middle series, white), and the target (right series, purple), for: **a** Algorithm #1, the baseline elementary motion detector (EMD) algorithm; **b** Algorithm #2, a more elaborated EMD algorithm including photoreceptor (PR) and lamina monopolar cells (LMC) pre-processing models; **c** Algorithm #3, the algorithm proposed in this research with the novel contrast adaptation disabled; and **d** Algorithm #4, the full proposed algorithm. Each algorithm features the same lobula plate tangential cell (LPTC) receptive field model for output responses. The LPTC outputs have been locally normalised for display purposes as the comparison between algorithms is based on statistical distribution of the responses using (23), not magnitude. The elaborations within the algorithm show a sequential increase in target discriminability, including the ability to estimate the time to impact to a plain-textured background distinctly differently to the target. As only the response vector magnitude was considered, and hence all responses will be greater than 0, the bidirectional noise that is typically present will produce an offset above 0, as shown in all tests

The results of the statistical analyses performed using Eq. (23) can be seen in Fig. 7. As each algorithm features the same LPTC receptive field model (e.g. the only spatial pooling function employed by our algorithm), Fig. 7 represents the comparison between the baseline (#1) algorithm and further elaborations (#2, #3, and #4). More detailed analyses of pooled outputs are presented later in Sects. 4.6 and  4.7. Boxes represent 75th (upper), 50th (median), and 25th (lower) percentiles of the data. Whiskers represent the 95th (upper) and 5th (lower) percentiles of the data. Outliers that are above and below these whiskers are also shown. The number of outliers varies as a function of the time to impact as there are a different number of samples in each set (see Sect. 3.4, specifically Table 2, for reasoning). The left box of each series (orange) represents the algorithm response to no motion and was taken as the mean of the last 50 frames of each full recording. The middle box of each series (white) represents the algorithm response to the background, and was taken as the mean response to frames 200 to 300. Finally, the right boxes (purple) represent the peak algorithm response to the target. Each recording has a full and a clipped version. The full recording includes 1 second (100 frames) of no motion at both the beginning and the end. The responses to no motion were taken from the end due to the presence of spurious initialisation outputs by the algorithm for the first N frames caused by the dynamic components of the algorithm adapting to the different scenes upon initialisation.

#### Algorithm #1

The first algorithm was a raw unelaborated EMD that calculated motion in both cardinal and ordinal directions and combined this with the LPTC receptive field used in this research. The energy estimate from the LPTC receptive field can be seen in Fig. 7a. The raw EMD expressed a statistical fitness of 0.888. As the contrast dependence of this model is well known, it was expected that a raw EMD would perform poorly when processing a diverse dataset. Additionally, the raw EMD is unable to statistically discriminate between the responses to no motion (left series, orange) and the background (middle series, white). Some discrimination can be seen for the peak target response (right series, purple), both compared to the no motion and background responses, as well as between times to impact. By inspection of the mean and median responses, it can be seen that the data is heavily positively skewed. That is, a higher proportion of the scenes have a lower-than-mean LPTC response.

#### Algorithm #2

The second algorithm built upon Algorithm #1 by adding the PR and LMC pre-processing stages, as well as the additional high-pass filtering to the EMD stage, while retaining the LPTC receptive field. The energy estimate from the LPTC receptive field can be seen in Fig. 7b. Unlike the raw EMD, the addition of the PR and LMC pre-processing stages has allowed the algorithm to begin to discriminate between the responses to no motion, the background, and the target. As such, the statistical fitness has increased to 1.786, a 201% increase over Algorithm #1. As the PR acts as a dynamic range compressor, contrast normaliser, and gamma corrector, the LPTC responses to the varying backgrounds now exhibit a largely normal distribution, which is consistent with literature (van Hateren 1997). The observed motion of the background is now also increased due to the increased local contrast produced by the PR and LMC stages.

#### Algorithm #3

The third algorithm built upon Algorithm #2 by including local motion adaptation and nonlinearity adjustments within the MLI model, while retaining the LPTC receptive field. The energy estimate from the LPTC receptive field can be seen in Fig. 7c. The statistical fitness of this algorithm was 3.427, a 192% increase over Algorithm #2, and a 386% increase over Algorithm #1. The addition of the motion adaptation and nonlinearity adjustments has allowed for greater statistical separation between the target and background responses. However, the extent to which the times to impact of the target are discriminable is still quite low. There was also minimal ability to distinguish between responses to no motion and the background.

#### Algorithm #4

Finally, the full algorithm proposed in this research builds upon Algorithm #3 by including a novel approach to contrast adaptation within the MLI model. The energy estimate from the LPTC receptive field can be seen in Fig. 7d. Unlike the previous algorithms, the proposed algorithm has excellent capabilities of discriminating between the target at the measured times to impact, with an overall statistical fitness of 14.910; a 435% increase over Algorithm #3, 834% over Algorithm #2, and 1679% over Algorithm #1. Not only is the target discriminable, a large response has also been obtained for the background (middle series, white). This is unexpected as the backgrounds are practically devoid of structure, apart from methodological errors such as a ripple in the fabric or local changes in luminance. The distance to the background is the distance to target, *L*, plus half the thickness of the target, 45 mm (see Sect. 3.1). The reduced relative distance between the background and target, plus the optical blur decreasing the perceived structure of the target, explains why the response to the background becomes less discriminable from the response to the target as the distance increases.

### Correlation of intensity rank ordering to algorithm output ordering

**Fig. 8:**
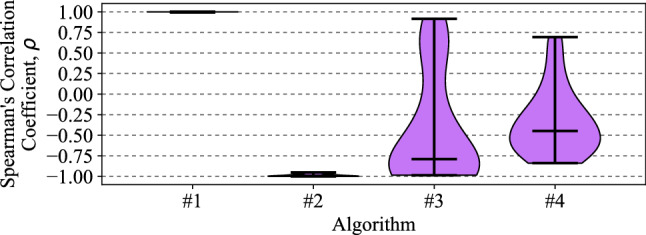
Spearman’s Correlation coefficient results for each algorithm comparing the rank order of the motion energy outputs against the rank order of the background intensities, where plot ticks represent median and extrema. As Algorithm #1 is a straight HR-EMD model, it exhibits a perfect correlation between energy output and intensity rank ordering. With the inclusion of the PR and LMC models prior to the HR-EMD in Algorithm #2, an inversion of the correlation is seen, where higher background intensities now produce lower energy outputs. Further elaborations to include the MLI and LPTC models to form Algorithm #3 show the beginnings of a generalisation of energy output, regardless of background intensity. Finally, the novel contrast adaptation used to form Algorithm #4 shows further reductions in energy output dependency upon background intensity. It is critical to note that this is merely the correlation between rank order of the algorithm outputs and background intensity; it lacks the context to infer relative *strength* of the dependence. For that, the other results in this paper must be considered

The correlation between the ranking of the background intensity, hence contrast between background and target, and the ranking of the algorithm outputs is shown in Fig. 8. Looking at the mean ± 95% confidence intervals, Algorithm #1 presents a very high positive correlation of 0.998 ± 0.001, Algorithm #2 a very high negative correlation of $-0.987$ ± 0.006, Algorithm #3 a low negative correlation of $-0.443$ ± 0.243, and finally Algorithm #4 a low negative correlation of $-0.336$ ± 0.151 (see Sect. 3.8 for further details).

### Conversion of LPTC output to time to impact

**Fig. 9:**
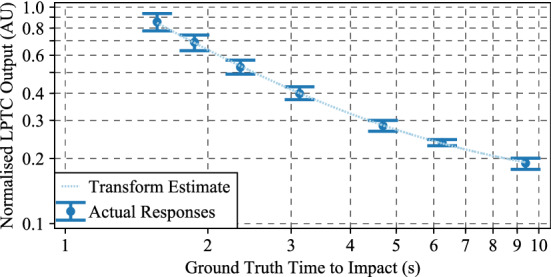
Output of the full algorithm proposed in this work, Algorithm #4, including the curve fit of the generalised logistic function from (18) used to transform the LPTC output into a time to impact estimate. The exact parameter values for the logistic function are less important than the ability of the logistic function to accurately represent the response characteristics. Actual responses are the same as Fig. 7. However, they are now represented on a log-log graph. Error bars are the 5th and 95th percentiles as used throughout the statistical analyses

Transforming the outputs of the LPTC (Fig. 7) into a time to impact estimate is achieved by performing a nonlinear least squares fit utilising (19). This equation was fit to the mean responses of each individual algorithm, and also considered the deviation (sigma) of the data points. The result of this curve fit against the actual LPTC outputs is shown in Fig. 9, where the LPTC responses for the full algorithm proposed in this work, Algorithm #4, and the curve fit estimates are shown.

The estimated time to impact results, as well as the ground-truth time to impact values, are shown in Table 4. As can be seen, as the equation has been fit to each algorithm, the mean time to impact estimates are relatively close to the ground truth. However, the distribution of the responses at each ground truth time to impact, and the lack of discriminability between disparate ground-truth times to impact, is reflected by the variance (±) in the time to impact estimate. This can also be seen in Fig. 7, where clear overlaps or separations between LPTC outputs are evident for each algorithm. As each algorithm has been individually tuned — that is, they have unique parameter sets and their outputs do not in any way relate to one another—it is not possible to utilise the transfer function of one algorithm to quantify the response or accuracy of another.

**Table 4:** Comparison between the ground-truth time to impact, as calculated from methodological set-up, and the time to impact after conversion of the motion energy output of each algorithm

Time to		**Algorithm**			
Impact (s)		**#1**	**#2**	**#3**	**#4**
1.563	$+\sigma$	NaN	0.983	1.159	1.438
	${\bar{x}}$	1.582	1.593	1.555	1.549
	$-\sigma$	NaN	3.173	2.877	1.679
1.875	$+\sigma$	NaN	1.158	1.401	1.745
	${\bar{x}}$	1.820	1.786	1.839	1.865
	$-\sigma$	NaN	3.852	3.273	2.019
2.344	$+\sigma$	NaN	1.522	1.769	2.212
	${\bar{x}}$	2.425	2.401	2.394	2.371
	$-\sigma$	NaN	4.643	3.681	2.546
3.125	$+\sigma$	NaN	2.049	2.392	2.913
	${\bar{x}}$	3.131	3.155	3.160	3.138
	$-\sigma$	NaN	6.360	4.576	3.363
4.688	$+\sigma$	NaN	3.154	3.680	4.381
	${\bar{x}}$	4.469	4.629	4.701	4.722
	$-\sigma$	NaN	9.877	6.104	5.119
6.251	$+\sigma$	NaN	4.130	4.691	5.854
	${\bar{x}}$	5.903	6.275	6.136	6.177
	$-\sigma$	NaN	14.206	8.172	6.521
9.376	$+\sigma$	NaN	6.208	6.733	8.473
	${\bar{x}}$	12.511	9.382	9.471	9.569
	$-\sigma$	NaN	22.528	17.358	11.518

Algorithm #1 is the baseline EMD model. Algorithm #2 improves upon #1 by inclusion of PR and LMC pre-processing models. Algorithm #3 improves upon #2 by inclusion of MLI and LPTC post-processing models, with the novel contrast adaptation within the MLI model disabled. Finally, Algorithm #4 is fundamentally equivalent to #3, with the novel contrast adaptation within the MLI model enabled. The addition of the standard deviation of the motion energy, $+\sigma$, corresponds to a decrease in the estimated time to impact, and the converse holds true for $-\sigma$

### Distribution of results by time to impact

**Fig. 10:**
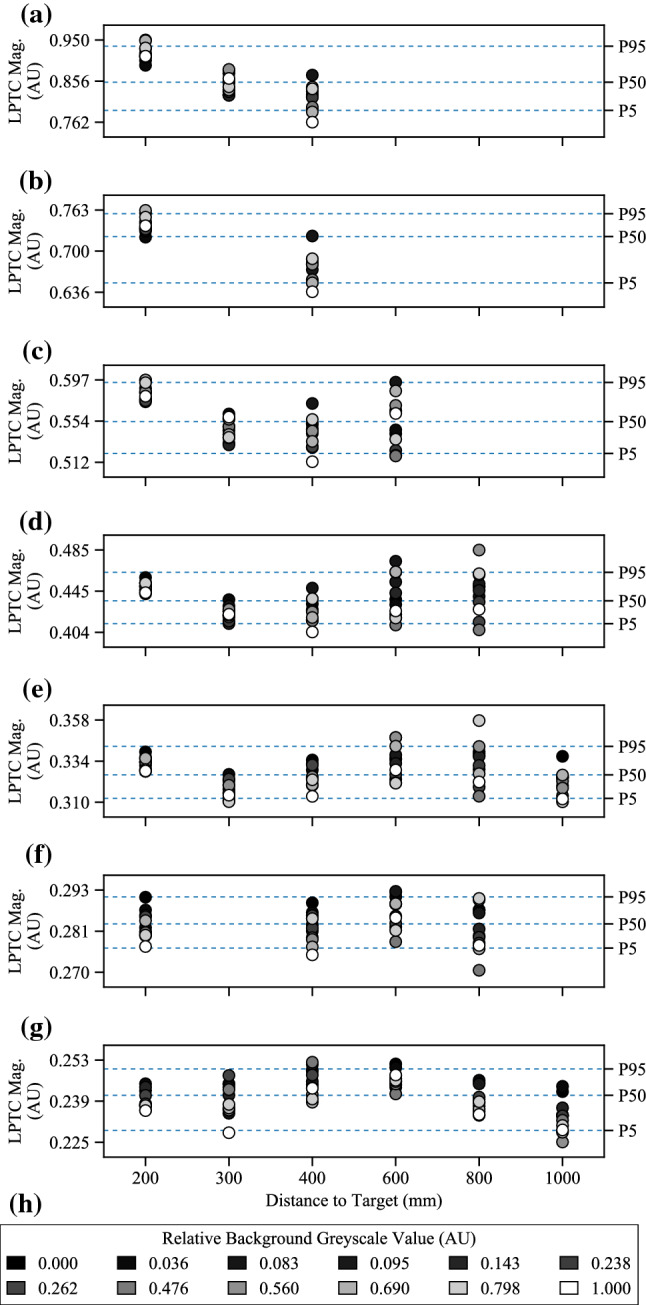
Algorithm response graphs separated by time to impact showing energy estimate variation based on distance to target for times to impact, $t_I$, of: **a** 1.563 s; **b** 1.875 s; **c** 2.344 s; **d** 3.125 s; **e** 4.688 s; **f** 6.251 s; **g** 9.376 s. **h** shows the distribution of relative background intensities. The different number of samples per time to impact is caused by not all distance and velocity pairs being present at all times to impact (refer to Sect. 3.4 for details). The lobula plate tangential cell (LPTC) model receptive field outputs have been normalised for display. Despite the varying distances to target, the distributions of the times to impact are statistically different. The statistical measure used (23) calculates based on the P5 (5th), P50 (50th, median), and P95 (95th) percentiles, which are indicated on the secondary y-axis and represented by horizontal dashed lines

Breaking down the results shown in Fig. 7d, the statistical distribution of the responses based on the time to impact can be seen in Fig. 10. No overlap was present between the statistical limits (5th and 95th percentiles) of any two time to impact groups. Variations between the responses to different speeds and distances indicate a complex relationship between the resolvable texture within the target and the system response. No clear pattern between target-to-background contrast and system response was observed.

At the shortest time to impact studied in this research, $t_I$ = 1.563 s, the responses (Fig. 10a) are as expected, where the closer distance to target has produced a higher LPTC magnitude output. This is likely caused by the increased visual structure detectable by the ommatidial resolution at the closest distance (*L* = 200 mm).

Looking at the next time to impact, $t_I$ = 1.875 s (Fig. 10b), a very similar distribution of the responses can be seen when compared to the previous one.

For the time to impact of $t_I$ = 2.344 s (Fig. 10c), the distribution is again similar, although the distribution of the responses at 600 mm to target is larger than those at closer distances. However, the distribution of the 600 mm responses does not fall outside the distributions of the previous distances to target.

At a time to impact of $t_I$ = 3.125 s (Fig. 10d), the responses for 200 mm to 400 mm distances are largely the same as the previous times to impact. At a distance of 800 mm, there is a single outlier on the high side of the LPTC responses. This outlier is likely caused by a change in environmental conditions, such as luminance or structure. Disregarding the outlier, the distribution of the 800 mm distance to target can be seen to exhibit a lower LPTC magnitude than that of the 600 mm distance to target.

For the time to impact of $t_I$ = 4.688 s (Fig. 10e), there are now samples from all distances to target. The response for distances from 200 mm to 800 mm is similar to those seen previously. The distribution of the LPTC magnitudes for a distance to target of 1000 mm continues to exhibit a lower magnitude than the previous times to impact.

For the time to impact of $t_I$ = 6.251 s (Fig. 10f), the distributions of all samples present are largely the same, apart from an outlier on the lower magnitude of the 800 mm sample. This distribution is different to the previous times to impact, where a clear decline in LPTC magnitude can be seen when moving from 200 mm to 300 mm. This is followed by an increase in LPTC magnitude through 400 mm, peaking at 600 mm. While this behaviour can be seen in the responses at this time to impact, the peak magnitudes are greatly reduced.

Lastly, the time to impact of $t_I$ = 9.376 s (Fig. 10g) again exhibits a different characteristic distribution of responses at the different times to impact. There is now a single clear peak around the 400 mm and 600 mm samples, with a single outlier on the 300 mm sample set. After the peak, the response exhibits lower LPTC magnitudes, which is to be expected.

These results show that there is a higher response at the closest range, likely caused by the increase in visual structure that is present at that distance and/or by the relative difference between the centre and outer edges of the object being larger. There is another peak in LPTC magnitude at 600 mm from target. The cause of this peak is unknown, and will be the subject of further research.

### Response to changing backgrounds

**Fig. 11:**
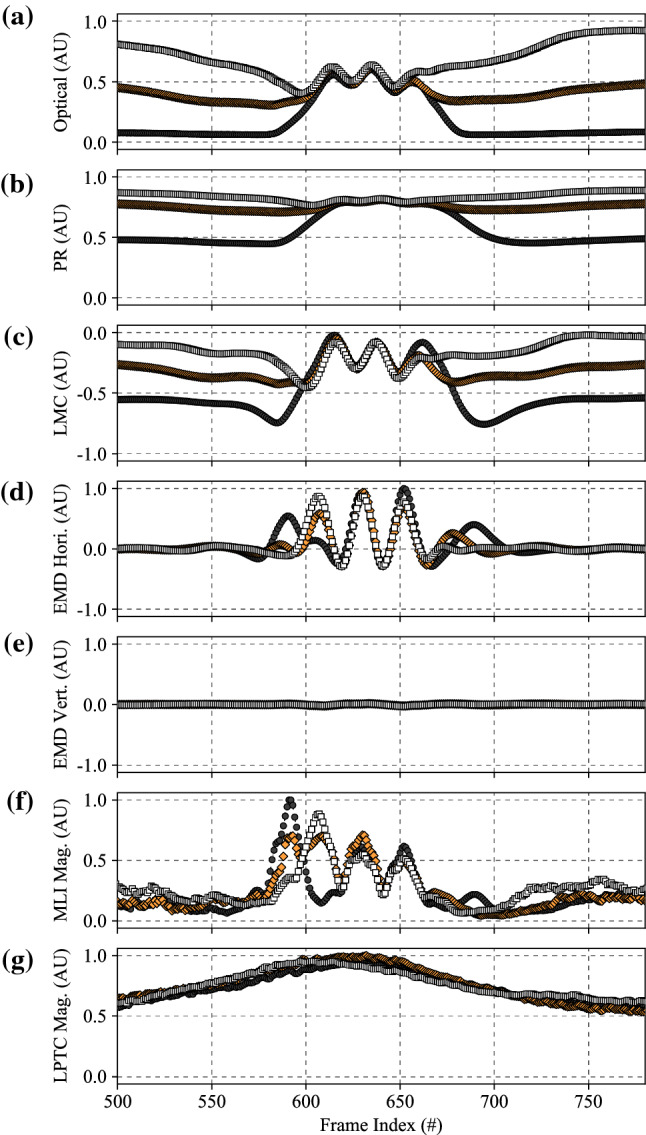
Region of interest responses for the output of each model within the proposed algorithm, against white (square markers), orange (diamond markers), and black (circle markers) backgrounds, for a distance of 200 mm and velocity of 127.98 mm/s ($t_{I}$ = 1.563 s): **a** The optical input to the algorithm; **b** The output of the photoreceptor (PR) model; **c** The output of the lamina monopolar cell (LMC) model; **d** The horizontal component of the output of the elementary motion detector (EMD) model; **e** The vertical component of the output of the EMD model, normalised the same as **d** to convey the lower signal magnitude of the vertical component; **f** The magnitude output of the medulla-lobula interneuron (MLI) model; and **g** The magnitude output of the lobula plate tangential cells (LPTC) model. All responses have been locally normalised for graphing purposes as their absolute signal magnitude is not of importance, just their statistical distribution. The LMC output (**c**) is not centred around 0 due to imperfect high-pass filtering. While the optical structure of the targets in (**a**) is similar, the responses of each model, especially the transition between background and target, are varied due to the drastic difference in background intensity, and hence contrast. Although the optical responses differ, the LPTC receptive field produces a very similar response for each background, although with some temporal misalignment present due to the adaptive filtering components of the algorithm. For best viewing, please see the online version of this article

The individual model responses for a target range of 200 mm, a velocity of 127.98 mm/s, and three different backgrounds, namely black (relative intensity 0.000), orange (relative intensity 0.476), and white (relative intensity 1.000), can be seen in Fig. 11. It shows the algorithm response after each separable model element, as depicted in Fig. 3. The final output of the model (Fig. 11g) is shown to be contrast independent; that is, the same response is observed regardless of the background colour.

Looking at the optical input responses (Fig. 11a), it can be seen that the structure of the target is relatively independent of the background colour, which is to be expected. The white background presents a slightly higher optical intensity for the target than the black and orange. However, this is expected as a white background is more reflective of environmental lighting than a darker background. This also explains the steeper slope of the intensity drop as the target comes into view, $i_{550}$ to $i_{600}$: the shadow caused by the target will be more pronounced on a brighter background. Similarly, when the target is exiting the FOV ($i_{650}$ to $i_{700}$), it can be seen that the white background is again at a higher intensity level than the target.

Next, the responses of the photoreceptors (PR’s) are shown in Fig. 11b. As the PR’s were adapted to the background for $\sim$6 seconds prior to the target entering the FOV, the PR’s have a more aggressive response to change in intensity introduced by the target in the dark backgrounds. This is caused by the PR’s being in a high-gain state with the lower intensity. After the initial transition from background to target ($\sim i_{600}$), the PR’s normalise their response to the structure of the target ($\sim i_{635}$) irrespective of the input intensity differences shown previously. Due to the various temporal components of the PR model, a frame delay of $\sim$6 frames, or $\sim$0.06 seconds at the 100.027 frames per second that the dataset was capture at, can be seen between the optical input and the PR outputs.

The responses of the lamina monopolar cells (LMC’s) can be seen in Fig. 11c. The purpose of the LMC is to remove redundant information and enhance intensity transitions. We can see the effect of the contrast enhancement when comparing the magnitude of the peaks caused by the target to that of the output of PR’s previously. All three key peaks have been amplified. However, the outputs of both stages are not comparable as $I_{PR} \in (0, 1.0]$, whereas $I_{LMC} \in [-1.0, 1.0]$. The amplitude offset present in the LMC is also less important as the high-pass filtering that is present within the next stage of processing, the EMD, is ideal; it removes all DC signal components, unlike the leaky high-pass filtering within the LMC.

Following the LMC’s are the elementary motion detectors (EMD’s), the responses of which are seen in Fig. 11d for the horizontal component and Fig. 11e for the vertical component. The fundamental principle of the EMD’s, which are modelled as Hassenstein–Reichardt detectors, is to correlate changes in incoming signal intensity between pixels. Therefore, the maximum response from an EMD will occur at the steepest part of a dark-to-light or light-to-dark transition produced by the preceding filtering. This causes the peak EMD responses to occur between the peaks of the target structure (outlines and texture), instead of at the boundaries. As the purpose of this research is to analyse the response to motion that is perpendicular to the system, the majority of optic flow that is present is horizontal. However, due to the angular misalignments during experimental set-up, angular components of the texture on the tree combined with the *aperture problem*, as well as noise that is present, some vertical component to the optic flow does exist. In practice, the magnitude of the vertical component was measured at $\sim$1% of the horizontal component. While this component is taken into account in the later stages of the algorithm, specifically the conversion from Cartesian to Polar coordinates, it has been graphed here at the same scale as the horizontal component to convey this reduction in magnitude. This generalisation to purely horizontal motion is only valid as the receptive field has been modelled perpendicular to the direction of optic flow. The vertical component will be critical when other receptive fields are used as they will deviate from this location.

The output of the EMD’s is processed by what we refer to as the medulla-lobula interneurons (MLI’s), the responses of which are shown in Fig. 11f. At this point, the horizontal and vertical outputs from the EMD have been converted from Cartesian (*x*, *y*) to Polar (*R*, $\theta$) representations; and this work focuses on the magnitude of the response, *R*. In this output, not only can the target contrast responses be observed, but the minor imperfections in the backgrounds are enhanced and can be observed as local contrast peaks which the system can use to track the optic flow even on uniform coloured cloth.

Finally, the output from the lobula plate tangential cells (LPTC’s) is seen in Fig. 11g. The effects of the large spatial pooling (receptive field) present in this model are evident when compared to the high frequency responses of the MLI model, as is the limited impact of any temporal filtering that also occurs. It is interesting to note the difference in peak response from this model compared to previous models, where the white background exhibits a peak response $\sim$30 frames prior to the black and orange backgrounds. This could potentially be caused by a higher-than-mean response of the white background (visible in the MLI at $i_{500}$) causing the receptive field to be at a higher state than the other backgrounds prior to the target entering the receptive field. The orange background shows a higher peak LPTC response overall, which corresponds to the similar response for the 3 peaks shown in the MLI model. The black background does have the highest magnitude MLI response ($\sim i_{590}$), which is most likely because it has the highest contrast difference between the background and the target. However, this quickly adapts away and results in a gap or decreased response ($\sim i_{610}$) which results in a very similar overall LPTC response to the other backgrounds. This behaviour will be the focus of ongoing work.

From these results, it has been shown that irrespective of the background, or more accurately the contrast between the background and the target, a consistent estimate of the time to impact to the target, as measured by the peak amplitude response, can be made. While there is a spatio-temporal misalignment between the peak LPTC responses of approximately 25 frames between the various backgrounds, this is unlikely to negatively impact a real-world application as it represents a time difference of at most 0.25 s in the responses. This time difference could be reduced by reducing the levels of adaptive filtering in the model, but that would result in larger response differences as the system would become more contrast sensitive in the output.

**Fig. 12:**
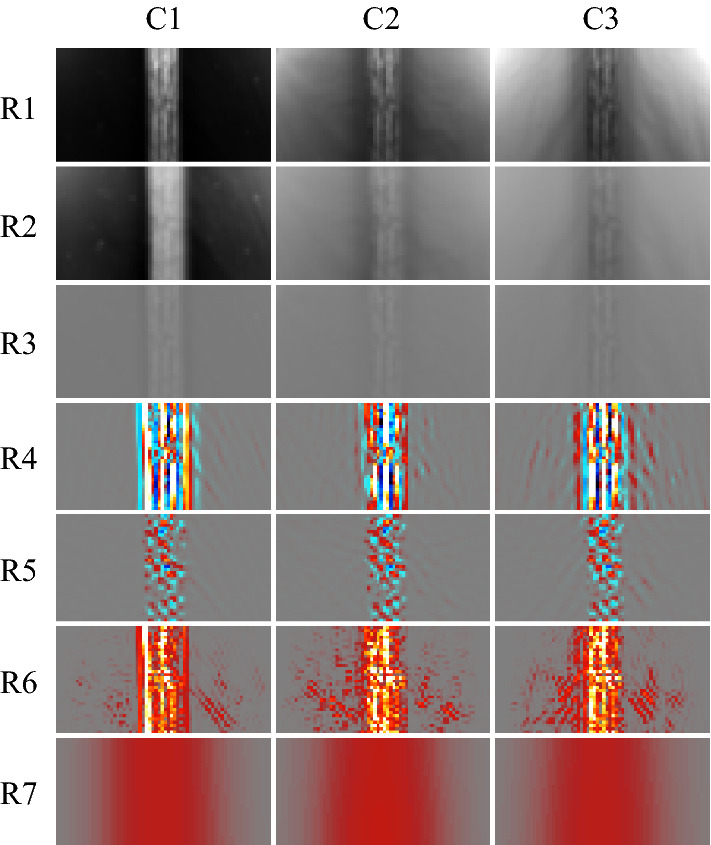
Component model responses at a distance of 200 mm, velocity of 127.98 mm/s ($t_I$ = 1.563 s), and for backgrounds: (C1) black; (C2) orange; and (C3) white. Model responses from processing frame index 0903 of the full recording have been rescaled, and the omnidirectional frame has been clipped to ±34$^\circ$ horizontal field of view around the left perpendicular, for display purposes. All exposure values and post-processing gains for display were equal across all scenarios. Each row represents a different stage of the algorithm: (R1) optical input to the photoreceptor (PR) model; (R2) output of the PR model; (R3) output of the lamina monopolar cell (LMC) model; R4) horizontal component of the output of the elementary motion detector (EMD) model; (R5) vertical component of the output of the EMD model, shown at the same scale as R4 where, due to the purely horizontal motion, the columns will typically sum to an amplitude of 0; (R6) the magnitude output of the medulla-lobula interneuron (MLI) model, which features the novel contrast adaptation proposed in this research; and (R7) the output of the lobula plate tangential cell (LPTC) model prior to the receptive field window being sampled. The imperfect high-pass filtering in the LMC model is evident by a lack of sharp definition in the edges of R3. The *aperture problem* associated with the EMD is strongly illustrated in R4 and R5 where deep blue and white hot are opposite extremes of a custom colour scale. The benefit of moving to polar coordinates in the MLI is shown in R6 where only positive magnitudes (approaching white hot) are present. Despite the drastic difference in optical inputs (R1), and different structures within the EMD responses (R4 and R5), the adapted output of the MLI model (R6) and the corresponding spatio-temporal blur of the LPTC (R7) show similar responses across the 3 backgrounds. These results are spatial representations of the temporal results shown in Fig. 11, where $\sim i_{630}$ corresponds to the responses shown here

The spatial responses of each model within the algorithm to the same backgrounds shown for the temporal responses (Fig. 11) are presented in Fig. 12. These outputs, for a distance to target of 200 mm and a velocity of 127.98 mm/s ($t_I$ = 1.563 s), are clipped to ±34$^\circ$ around the left perpendicular to remove redundant/distracting environmental information from the outputs. It can be seen that there is a perceptibly large difference in the optical representation of the target against the background as the intensity of the background changes (R1). The PR model aids in somewhat normalising the input intensity (R2) and, although it is imperfect, the high-pass filtering present within the LMC model is demonstrated (R3). Recall that stronger (perfect) high-pass filtering is present within the EMD model. The *aperture problem* associated with the EMD model is demonstrated (R4, R5), where the directionality of the optic flow is swinging both sides of 0, with a custom colour profile (black/blue/cyan and yellow/red/white) representing opposite directions of optic flow. While there is also some vertical motion present (R5), this is a much lower magnitude than the horizontal energy (R4). To aid in overcoming the *aperture problem*, the MLI operates in polar coordinates (R6), specifically utilising only the magnitude of the response vector. The addition of contrast and motion adaptation with the MLI can be seen, where there is not only a more consistent response to the target (central), but also a response to the background. Once the spatio-temporal blur of the receptive field of the LPTC model is applied (R7), the responses are similar for each background shown.

### Response to changing distance

**Fig. 13:**
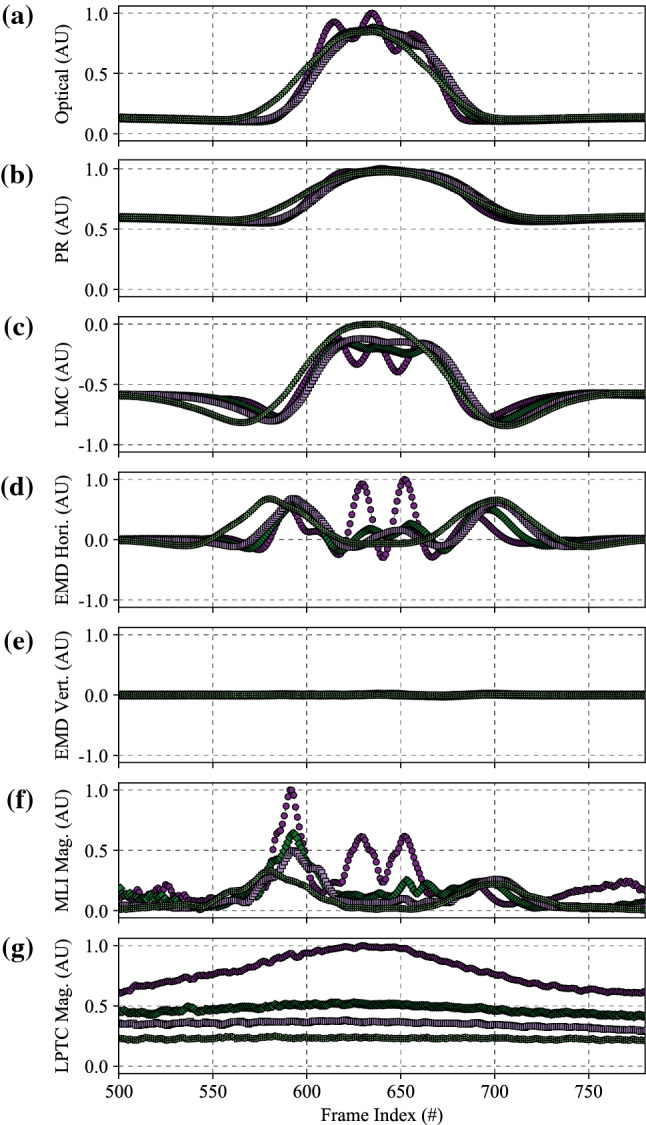
Region of interest responses for each model within the proposed algorithm for a black background at a velocity of 127.98 mm/s, for distances of 200 mm ($t_{I}$ = 1.563 s, dark purple, circle markers), 300 mm ($t_{I}$ = 2.344 s, dark green, diamond markers), 400 mm ($t_{I}$ = 3.125 s, light purple, square markers), and 600 mm ($t_{I}$ = 4.689 s, light green, plus markers): **a** Optical input; **b** Output of the photoreceptor (PR) model; **c** Output of the lamina monopolar cell (LMC) model; **d** Horizontal output of the elementary motion detector (EMD) model; **e** Vertical output of the EMD model, normalised per (d) to reflect the lower signal magnitude of the vertical component; **f** Output of the medulla-lobula interneuron (MLI) model; and **g** Output of the lobula plate tangential cell (LPTC) model. All responses were locally normalised as their absolute signal magnitude is not of importance, just their statistical distribution. The LMC output **c** is not centred around 0 due to imperfect high-pass filtering. Despite similarities between the peak amplitudes at the optical input, the LPTC is able to distinguish between the different times to impact caused by the different distances to the target. At the larger distances, the response to the target becomes less distinguishable compared to the background (see Fig. 7d). The different LPTC responses at the beginning (i500) and end (i775) are due to the different distances to the background; that is, the model is tracking the time to impact to the background, which is almost indistinguishable from the target at farther distances. For best viewing, please see the online version of this article

Figure 13 shows the input–output characteristics of the responses of the various models within the proposed algorithm as the distance to target, and hence the time to impact, changes while the translational velocity is constant. In this figure, the velocity was constant at 127.98 mm/s, the background was black, and the distances to target were 200 mm ($t_{I}$ = 1.563 s, dark purple, circle markers), 300 mm ($t_{I}$ = 2.344 s, dark green, diamond markers), 400 mm ($t_{I}$ = 3.125 s, light purple, square markers), and 600 mm ($t_{I}$ = 4.689 s, light green, plus markers). Although there appears to be similarity in the peak magnitude of the optical input at each distance, and hence time to impact, the algorithm is capable of discriminating the time to impact of each distance.

Looking at the optical input to the PR (Fig. 13a), there is a clear reduction in the perceivable structure of the target as the distance increases. The response at 200 mm clearly portrays the 3 stages of the target: the initial transition from background to target ($\sim i_{615}$); the central structure within the target ($\sim i_{635}$), and the final transition from the target back to the background ($\sim i_{660}$). As the distance increases, this structure is lost primarily due to the low ommatidial resolution of 180 pixels for 360$^\circ$ horizontal FOV combined with the anti-aliasing optical blur employed after the dewarping process prior to resolution reduction. While there is a loss of registration of the structure, the peak magnitude of the target ($\sim i_{635}$) remains relatively similar as the distance increases, which is expected since it is optically the same target. Due to the spatial distance realised by each pixel increasing as the distance to target increases, combined with the optical blur, the target appears wider as the distance increases. So, while the peak magnitude remains similar, the transition point from background to target appears earlier, and target back to background appears later, as the distance to target increases. As the same background was used for these tests, the optical responses to the background are virtually identical.

In the output of the PR model (Fig. 13b), it can be seen that the PR model has nearly normalised the intensity of the input signal, most evident by the smoothing of the response to the 200 mm distance.

Next, in the output of the LMC model (Fig. 13c) the edge enhancement functionality of the LMC’s is evident. While the PR had worked to normalise the input intensity, the LMC serves to remove redundant information and to enhance changes in intensity; in other words, edge enhancement. The large downward responses at the transition points between background and target are caused by the reduction in intensity in these regions caused by the shadows, most noticeable on the methodological set-up shown in Fig. 2. Light to dark transitions are seen as negative responses, while dark to light transitions are positive responses.

The horizontal component of the EMD model motion energy estimation (Fig. 13d) conveys the nature of the EMD; the estimation of motion energy is directly proportional to the change in intensity of the input signal.

While there is motion estimated by the vertical component of the EMD model (Fig. 13e), when normalised to the range of the horizontal component, the relative insignificance of the vertical component becomes apparent, where signal strength is $\sim$1% of the horizontal component. This is, of course, due to the location that the receptive field is sampled from: perpendicular to the direction of travel at the horizon. The vertical component becomes larger, and more relevant, the further dorsal or ventral the receptive field is located, and also the further away from the horizon.

The magnitude output of the MLI model (Fig. 13f), which features the novel contrast adaptation, shows the behaviour when the model transitions from a high-gain state caused by a black background, to the target response ($\sim ~i_{590}$). Once adapted to the new conditions presented by the target, the magnitude of the responses across all distances attenuates. While the target previously presented similar magnitudes for leading and trailing edges in previous progressing stages, this is no longer the case with the trailing edge of the target much smaller than the leading edge in all cases.

Finally, the spatial pooling functionality of the LPTC model (Fig. 13f) is easily seen with a relative smoothing of the motion energy estimated by the MLI model. Additionally, the rapid temporal aspect of the spatio-temporal adaptation is evident, as the peak response to the target roughly corresponds to the peak magnitudes of the optical input. That is to say, no significant spatial offset of the true location of the target has been introduced by the use of spatio-temporal adaptation. In the context of using this algorithm as a navigational tool, specifically for obstacle avoidance, this allows for increased certainty in the location of a detected obstacle, allowing for more accurate navigational decisions to be made.

## Discussion

The results presented in this work show that time to impact of targets is differentiable between, for example, 1.5 seconds and 1.8 seconds. However, in reality, it is unlikely that a navigational control system of a robotic system, which has been extensively researched for many years (Khatib 1985, 1986; Borenstein and Koren 1989, 1991), would want to act on this resolution. Additionally, this work has only considered the magnitude of the response vectors in the MLI model. The benefit of this is that it produces an instantaneous estimate of time to impact between the camera (e.g. vehicle) and the environment, regardless of the directionality of movement of one or the other. Future work will look at integrating the direction component as this will be required when used as a navigational cue.

A key aspect of a biologically inspired algorithm is the ability to tune it. For example, although several insects express extreme accelerations and velocities during locomotion, that level of response is less important to a large, slow moving autonomous vehicle. Previous work on the low-speed rotational velocity estimation component of inter-saccadic motion (Skelton et al. 2017, 2019) has shown that the operating range can be tuned based on the platform being used (Skelton et al. 2020). While the work presented in this paper has demonstrated the performance of the algorithm in the operating range of 1.5 seconds to 9.3 seconds, there is no particular reason why this range could not be expanded to 1.5 seconds to 93.0 seconds, at the cost of resolution between those boundaries. Likewise, it is possible to shift the operating range to longer time to impacts, at the cost of introducing saturation and/or aliasing at shorter time to impacts. This environmental adaptation, or contextual tuning, is also expressed in biology (Arenz et al. 2017).

One of the peculiar aspects of the proposed algorithm is the separate spatial high-pass filters present towards the end of the LMC model, and as the first operation of the elaborated EMD model (see Fig. 3a). Although there is a constant gain applied to the output of the LMC model, this is a linear transform and thus does not impact the behaviour of the subsequent high-pass filter. The justification for this separability is that parallel processing pathways in biology diverge from the motion estimation pathways between the LMC and the EMD. For example, the small target detection and tracking pipelines have different input signal requirements to the EMD (Melville-Smith et al. 2019). Thus, the algorithm has been configured in such a way as to facilitate compatibility with parallel processing pipelines.

While recent literature in neurophysiology has demonstrated that contrast adaptation of insects is primarily a temporal function (Matulis et al. 2020), the work presented here challenges that by demonstrating that spatio-temporal contrast adaptation, with an emphasis on the spatial processing and little temporal processing, can be extremely beneficial. The results of rank ordering analysis, presented in Sect. 4.3, shows that great improvements have been made to overcoming the contrast dependence of algorithms based upon the HR-EMD. While a low negative correlation still exists for Algorithm #4, this must be taken in context with the discriminability capabilities of the algorithm, as highlighted in Fig. 10. It would be entirely possible to create an algorithm that is not intensity rank order dependent, but is incapable of discriminating between times to impact. This work shows that both are possible.

This difference between biological recordings and technological solutions is a well-known problem, and is characteristic of neither system being fully explored and understood. It is entirely possible that there are spatial aspects to biology that have thus eluded neurophysiological investigations. This is possibly the case as it has been shown (Drews et al. 2020; Carandini and Heeger 2012) that biology handles contrast adaptation by an ON–OFF pathway split. This may help to explain why neurophysiologists observe largely temporal responses to contrast changes, as the responses driven by spatial processing would precede the ON–OFF pathway divergence.

Conversely, it is also entirely possible that the technological implementations of the biological systems are not 100% biomimetic. That is, they do not faithfully mimic the processes involved in biology. Realistically, both possibilities are true; not enough of the biological system is understood to faithfully reproduce it in a technological solution. However, this work does hint at the possibility of spatial processing that could be an exciting avenue of future neurophysiological investigations.

Unlike previous research that focussed on the low-speed rotational velocity estimation required for maintaining a heading during inter-saccadic movements (Skelton et al. 2017, 2019), the research presented in this paper was not intended to achieve a real-time real-world implementation. Several inefficiencies exist within the implementation, such as computationally expensive division operations used for the optical scaling (see Sect. 3.2.4) where multiplications could be used instead, or the spatial pooling of the LPTC being applied to the entire optical field rather than the receptive field being targeted. Nevertheless, frame rates in excess of 500 frames per second were achieved on consumer grade desktop hardware (4-core, 8-thread Intel Core i7-4790 with 8GB system memory); a fundamental benefit of working with ommatidial resolutions, where it has been demonstrated that significant sensory information can be derived from very low resolution inputs (Brinkworth and O’Carroll 2009). The real-time ability of the proposed algorithm, in combination with the parallel processing pathways of the insect visual system, will be the focus of future work.

In this work, only one perpendicular receptive field was modelled for analysis. In reality, insects utilise a variety of receptive fields at different locations and scales to perform different tasks. There are several possible avenues for addressing this expansion in information collection, such as utilising the expected unit vectors at a given location to scale the optic flow received. This approach, and several others, will also be the focus on future work.

## Conclusion

This paper has shown a novel approach for obtaining contrast independence for time to impact calculation during translational motion. By applying biologically inspired processing, statistical discrimination of 15 points per decade has been achieved within the operating range of 1.3 seconds through to 9.3 seconds of time to impact. This range was chosen as contextually for an autonomous ground vehicle it was deemed to be an acceptable amount of reaction time to cease locomotion or undertake avoidance manoeuvres. However, the algorithm can easily be adapted to other operating ranges by manipulation of the various temporal, spatial, and spatio-temporal filters. This work acts as a continuation of the low-speed rotational component of saccadic motion reported previously in (Brinkworth and O’Carroll 2009; Skelton et al. 2017, 2019), and provides another step towards true real-time operation of the complex movement behaviour of insects in biology. With a plausible approach to overcoming the contrast dependence of time to impact estimations presented, work can now continue on optimisation of the approach to facilitate high frame rate real-world, real-time deployments on embedded systems.

## Data Availability

The dataset captured for this research, in raw Polar format (size $\approx$ 539 GB), is available from the corresponding author on reasonable request. As the extensive source code is part of a much larger software ecosystem that is currently under commercialisation efforts, it is not available for public distribution.
